# *In Vivo* Approaches Reveal a Key Role for DCs in CD4+ T Cell Activation and Parasite Clearance during the Acute Phase of Experimental Blood-Stage Malaria

**DOI:** 10.1371/journal.ppat.1004598

**Published:** 2015-02-06

**Authors:** Henrique Borges da Silva, Raíssa Fonseca, Alexandra dos Anjos Cassado, Érika Machado de Salles, Maria Nogueira de Menezes, Jean Langhorne, Katia Regina Perez, Iolanda Midea Cuccovia, Bernhard Ryffel, Vasco M. Barreto, Cláudio Romero Farias Marinho, Silvia Beatriz Boscardin, José Maria Álvarez, Maria Regina D’Império-Lima, Carlos Eduardo Tadokoro

**Affiliations:** 1 Instituto de Ciências Biomédicas, Universidade de São Paulo, São Paulo, Brasil; 2 Instituto Gulbenkian de Ciência, Oeiras, Portugal; 3 Medical Research Center, London, United Kingdom; 4 Departamento de Biofísica, Escola Paulista de Medicina, Universidade Federal de São Paulo, São Paulo, Brasil; 5 Departamento de Bioquímica, Instituto de Química, Universidade de São Paulo, São Paulo, Brasil; 6 Unité d’Immunologie et Neurogénétique Expérimentales et Moléculaires (CNRS—UMR7355), Université d’Orléans, Orléans, France; Faculdade de Medicina da Universidade de Lisboa, PORTUGAL

## Abstract

Dendritic cells (DCs) are phagocytes that are highly specialized for antigen presentation. Heterogeneous populations of macrophages and DCs form a phagocyte network inside the red pulp (RP) of the spleen, which is a major site for the control of blood-borne infections such as malaria. However, the dynamics of splenic DCs during Plasmodium infections are poorly understood, limiting our knowledge regarding their protective role in malaria. Here, we used *in vivo* experimental approaches that enabled us to deplete or visualize DCs in order to clarify these issues. To elucidate the roles of DCs and marginal zone macrophages in the protection against blood-stage malaria, we infected DTx (diphtheria toxin)-treated C57BL/6.CD11c-DTR mice, as well as C57BL/6 mice treated with low doses of clodronate liposomes (ClLip), with *Plasmodium chabaudi* AS (*Pc*) parasites. The first evidence suggesting that DCs could contribute directly to parasite clearance was an early effect of the DTx treatment, but not of the ClLip treatment, in parasitemia control. DCs were also required for CD4+ T cell responses during infection. The phagocytosis of infected red blood cells (iRBCs) by splenic DCs was analyzed by confocal intravital microscopy, as well as by flow cytometry and immunofluorescence, at three distinct phases of *Pc* malaria: at the first encounter, at pre-crisis concomitant with parasitemia growth and at crisis when the parasitemia decline coincides with spleen closure. *In vivo* and *ex vivo* imaging of the spleen revealed that DCs actively phagocytize iRBCs and interact with CD4+ T cells both in T cell-rich areas and in the RP. Subcapsular RP DCs were highly efficient in the recognition and capture of iRBCs during pre-crisis, while complete DC maturation was only achieved during crisis. These findings indicate that, beyond their classical role in antigen presentation, DCs also contribute to the direct elimination of iRBCs during acute *Plasmodium* infection.

## Author Summary

Malaria is a significant health issue, particularly in the tropical and subtropical regions of the world. The red pulp (RP) of the spleen is a major site for the control of blood-borne infections such as malaria. Macrophages and dendritic cells (DCs) form a complex phagocyte network inside the splenic RP. DCs are usually thought of as highly efficient antigen-presenting cells that play an essential role in the activation of adaptive immunity. However, the direct role of DCs in the clearance of pathogens is still unclear. To clarify these issues, we took advantage of *in vivo* experimental approaches that enabled us to deplete or visualize DCs. The depletion of phagocytes demonstrated that DCs are key participants in the protection against blood stages of experimental malaria. Using confocal intravital microscopy, we observed that splenic RP DCs efficiently recognized and phagocytized infected erythrocytes during acute infection. We also showed that splenic DCs were crucial for the CD4^+^ T cell response to infection, but full DC maturation was achieved only after the peak of parasitemia. This study help to elucidate the protective mechanisms against *Plasmodium* parasites, and it shows that *in vivo* imaging is a reliable tool to evaluate iRBC phagocytosis during experimental malaria.

## Introduction

The spleen is a primary site for the control of blood-borne infectious diseases in humans and rodents [1], [2]. Although splenic phagocytic activity has been well documented *in vitro*[3]-[5] and *ex vivo* [3], [6]-[8], few studies have reported on the *in vivo* three-dimensional (3D) interactions between splenic phagocytes and pathogens [9]. Addressing this issue is particularly important in the case of malaria, a disease characterized by splenic involvement that is critical for controlling blood-stage *Plasmodium* parasites [10]. In recent years, confocal intravital microscopy (CIVM) [11] has been used to study host-pathogen interactions during infectious diseases caused by viruses [12], [13], bacteria [14] and protozoan parasites [15]. For example, CIVM revealed important aspects of the *Plasmodium* life cycle [16], [17]. Other works described *Plasmodium*-induced immune responses inside the placenta [18] and the dermis using fluorescent stereomicroscopy [19]. A single publication reported the movements of *Plasmodium*-infected red blood cells (iRBCs) inside the spleen [20]. However, no *in vivo* study has addressed the interactions between blood-stage *Plasmodium* parasites and the splenic immune system.

Splenectomized patients with acute *Plasmodium falciparum* infections have an impaired ability to remove parasites from circulation [21], similar to splenectomized mice infected with the blood-stages of *Plasmodium chabaudi* (*Pc*) [22]. In humans and mice, the phagocytosis of iRBCs or free merozoites by splenic phagocytes begins soon after infection and helps to control the parasitemia and induce the lymphocyte response [23], [24]. This occurs primarily inside the red pulp (RP) and the marginal zone (MZ) of the spleen [23], [24], where a complex phagocyte network is formed by heterogeneous populations of macrophages and dendritic cells (DCs) [25], [26]. In an effort to characterize the role of splenic phagocytes in *Pc* malaria, a recent study identified migrating monocytes as major participants in the clearance of iRBCs [8]. However, previous studies that quantified the *ex vivo* phagocytosis of iRBCs by flow cytometry reported low percentages of splenic phagocytes containing *Pc* remnants [8], [27]. This observation is not fully compatible with the notion that the role of the spleen is of the utmost importance in parasite control.

DCs are phagocytes that are highly specialized in presenting antigens to T cells [28]. Splenic DCs are efficient antigen presenting cells (APCs) during the massive T and B cell responses to acute *Pc* malaria [29]-[31]. Within the first week of *Pc* infection, splenic DCs up-regulate the expression of major histocompatibility complex (MHC) and costimulatory molecules, secrete pro-inflammatory cytokines, and stimulate T cell proliferation and IFN-γ production [32]-[34]. Nevertheless, it is still unclear whether DCs are unique in their ability to initiate CD4^+^ T cell responses to *Pc* blood-stages in the spleen, as observed in *Plasmodium berghei*(*Pb*) malaria [35]. Moreover, many details concerning the dynamics of splenic DCs in malaria remain unknown, limiting our understanding of the involvement of these cells in the protective immune response. After taking on antigens, immature DCs lose the ability to phagocytize and migrate towards T cell-rich areas to initiate the adaptive immune response [28]. Thus, it would be expected that DCs leave the RP soon after phagocytizing iRBCs or free merozoites and no longer contribute to parasite clearance, although this is as yet only a supposition.

In this study, we took advantage of experimental approaches that enabled us to deplete or visualize splenic DCs *in vivo* to clarify these issues. The *in vivo* depletion of phagocytes clearly demonstrated that DCs are key participants in the early control of the blood stage of infection with *Pc* and *Plasmodium yoelii* (*Py*) iRBCs, as well as the blood stage of infection with *Pb* sporozoites. The phagocytosis of *Pc* iRBCs by splenic DCs was analyzed by CIVM, as well as by flow cytometry and immunofluorescence, in three distinct situations: at the first encounter, at a pre-crisis phase concomitant with parasitemia growth and at a crisis phase, when parasitemia has dramatically dropped and changes in the splenic architecture have culminated in spleen closure [36]. CIVM allowed us to visualize the phagocytosis of *Pc* iRBCs by the RP DC network, the movement dynamics and morphological changes of DCs and the interaction between DCs and CD4^+^ T cells at the different phases of acute *Pc* malaria. To our knowledge, this is the first description of the *in vivo* interaction between *Plasmodium* iRBCs and the splenic immune system.

## Results

### DCs are required for parasitemia control and splenic CD4^+^ T cell activation during the blood stage of experimental malaria

**Figure 1:**
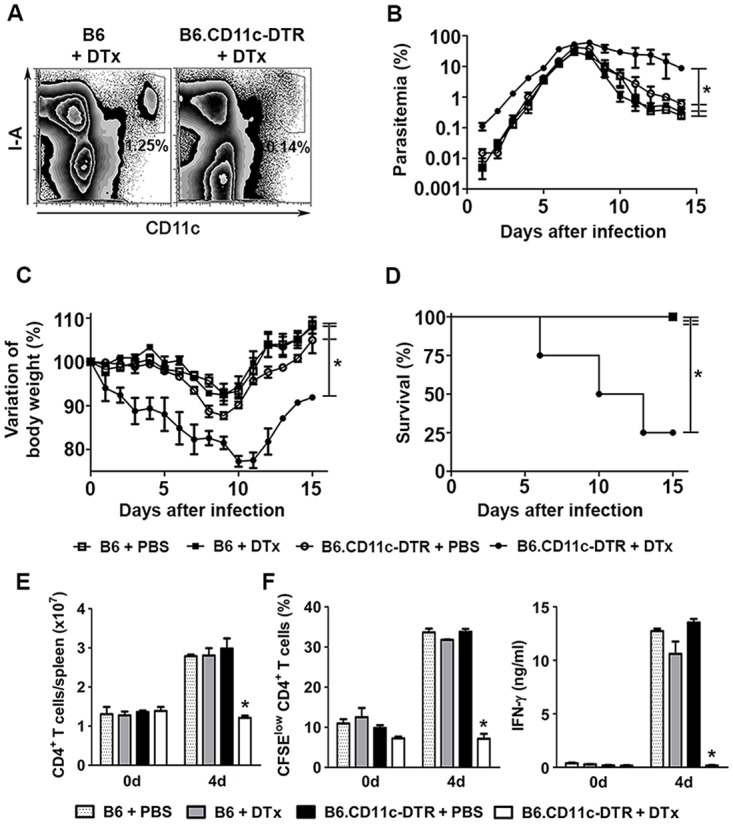
Effects of DC depletion on acute *Pc* malaria. **(A-F)** B6 and B6.CD11c-DTR mice were treated with either DTx to deplete CD11c^+^ cells or PBS as a control. The mice were i.p. infected with 1 × 10^6^ *Pc* iRBCs 24 h later. **(A)** Representative contour plots obtained 24 h after treatment by flow cytometry confirm the efficiency of DTx-induced depletion of splenic CD11c^+^I-A^+^ cells in B6.CD11c-DTR mice. Data show the percentages of CD11c^+^I-A^+^ cells in the splenocyte population. **(B)** Parasitemia curves are shown (means ± SD). **(C)** Variations in body weight relative to day 0 are shown (means ± SD). **(D)** Survival curves are shown. **(E)** Data show the numbers of CD3^+^CD4^+^ T cells per spleen at days 0 and 4 p.i. (means ± SD). **(F)** Data show the percentages of proliferating CFSE^low^CD4^+^ T cells and IFN-γ concentrations in the supernatants of spleen cell cultures stimulated for 72 h with iRBCs (means ± SD). In **B-D**, significant differences (p < 0.05) between the indicated groups are designated by *. In **E** and **F**, significant differences (p < 0.05) between all other groups are designated by *. In **A-F**, one representative experiment out of three (n = 5) is shown.

To evaluate whether DCs are important for the early control of blood-stage *Pc* malaria, C57BL/6.CD11c-DTR (B6.CD11c-DTR) mice were treated with diphtheria toxin (DTx). The great majority of splenic CD11c^+^I-A^+^ cells were eliminated in DTx-treated B6.CD11c-DTR mice (Fig. 1A). No effect was observed on F4/80^+^ RP macrophages, but the already small population of MARCO/MOMA-1^+^ MZ macrophages was depleted (S1 Fig.). Starting in the earliest days of infection, DTx-treated B6.CD11c-DTR mice had higher parasitemia (Fig. 1B) and weight loss (Fig. 1C) in comparison to their PBS-treated counterparts, leading to an accumulated mortality of 75% of mice on day 15 p.i. (Fig. 1D). On day 4 p.i., DTx-treated B6.CD11c-DTR mice had reduced numbers of CD4^+^ T cells per spleen (Fig. 1E). DTx treatment also completely abrogated the CD4^+^ T cell proliferation and IFN-γ production *in vitro* in response to iRBCs (Fig. 1F). None of these effects were observed in DTx-treated C57BL/6 (B6) mice (Figs. 1 and S1). Furthermore, the selective elimination of MZ macrophages by treating B6 mice with a low dose of clodronate liposomes (ClLip) did not affect the course of parasitemia, IFN-γ production by splenic CD4^+^ T cells or mouse survival (S2 Fig.). Similarly to what was observed for the *Pc* parasite, DTx treatment in B6.CD11c-DTR mice exacerbated *Py* malaria from the beginning of infection (S3A–S3C Fig.). The role of DCs in the early control of parasitemia was also evaluated in B6 and B6.CD11c-DTR mice that were treated with DTx on day 2 p.i. with *Pb* sporozoites. DTx-treated B6.CD11c-DTR mice presented with higher parasitemias (S3D–S3E Fig.). In this case, however, DTx treatment prolonged the survival of infected B6.CD11c-DTR mice by protecting them from cerebral malaria (S3F Fig.).

### Splenic DCs rapidly phagocytize iRBCs in recently infected mice

**Figure 2:**
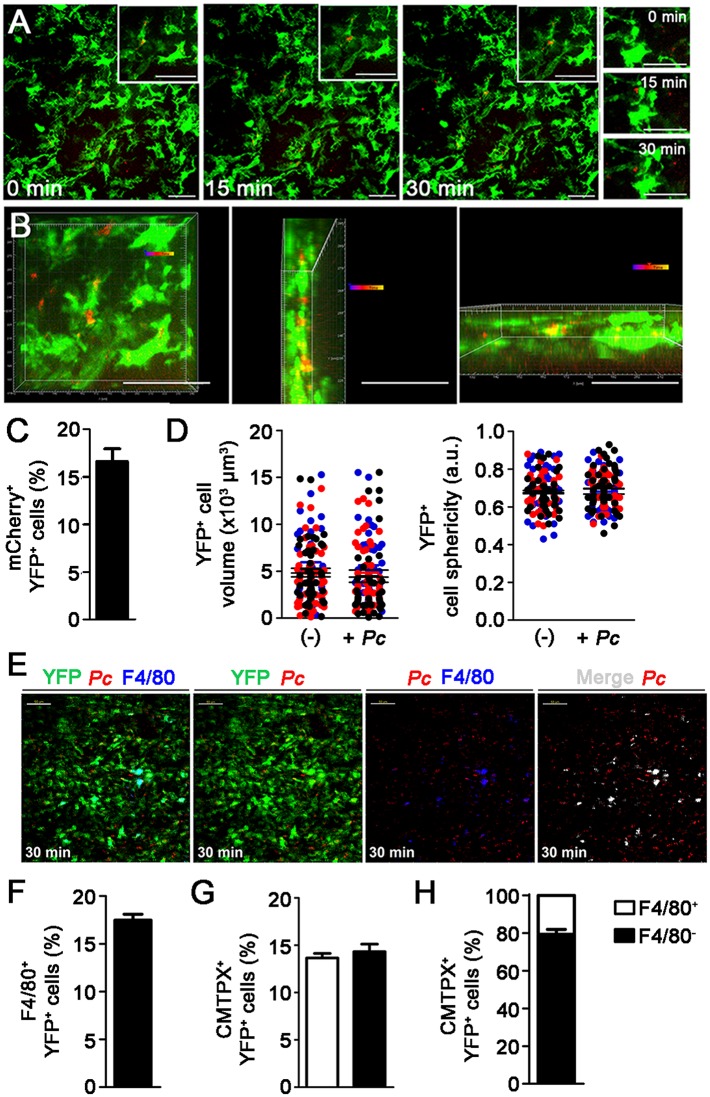
*In vivo* analysis of iRBC uptake by subcapsular RP DCs soon after *Pc* infection. **(A-D)** B6.CD11c-YFP mice were i.v. infected with 1 × 10^8^ mature mCherry-*Pc* iRBCs. Spleens were analyzed by CIVM after 15 min and in non-infected controls. **(A)** Serial snapshots taken at 15 min p.i. (0 min) show the subcapsular RP. The upper panels show the amplification of a few cells co-expressing YFP (green) and mCherry (red). In the image on the right, a region showing contact between YFP^+^ cells and mCherry-*Pc* iRBCs is magnified. **(B)** CIVM 3D animation reveals that YFP^+^ cells contain mCherry-*Pc* iRBC remnants (yellow spots of merged mCherry/YFP-3D signal). **(C)** Percentage of mCherry^+^ cells in the YFP^+^ cell population is shown (mean ± SEM). **(D)** YFP^+^ cell volume and sphericity are shown for naïve mice (-) and recently infected mice (+*Pc*). Black, red and blue dots are from three different experiments. Horizontal lines represent mean values and SEM. **(E**-**H)** B6.CD11c-YFP mice were i.v. infected with 1 × 10^8^ mature CMTPX-*Pc* iRBCs. After 15 min, mice were injected i.v. with a fluorescent anti-F4/80 mAb and the spleens were analyzed by CIVM. **(E)** Snapshots taken 30 min later show YFP^+^ cells (green), iRBCs (red), F4/80^+^ cells (blue) and merged F4/80^+^YFP^+^ cells (white) in the subcapsular RP. **(F)** Percentage of F4/80^+^ cells in the YFP^+^ cell population is shown (mean ± SEM). **(G)** Percentages of CMTPX^+^ cells in the F4/80^+^YFP^+^ and F4/80^-^YFP^+^ cell subsets are shown (means ± SEM). **(H)** The relative proportions of F4/80^+^ and F4/80^-^ cells in the CMTPX^+^YFP^+^ cell population were calculated from the data obtained in **F** and **G** (means ± SEM). In **A, B** and **E**, the scale bars correspond to 50 µm. One representative experiment out of three (n = 2) is shown. In **C, D, F, G** and **H**, data were calculated using Imaris software. Data from three experiments (n = 2) are shown.

To investigate whether splenic DCs phagocytize iRBCs in recently infected mice, we analyzed the interaction between YFP^+^ cells and mCherry-*Pc* iRBCs in the subcapsular RP of C57BL/6.CD11c-YFP (B6.CD11c-YFP) mice using CIVM [26]. Mice were infected by i.v. administration of mature iRBCs (>95% late trophozoites/schizonts), as these cells are known to be recognized and phagocytized by DCs [37]. In naïve mice, YFP^+^ cells were non-motile and actively extended protrusions and dendrites (S1 Video). At 15 min p.i., mCherry-*Pc* iRBCs were present in the subcapsular RP (Fig. 2A, S2 Video). CIVM 3D animations showed mCherry-*Pc* iRBC remnants inside YFP^+^ cells (yellow spots of merged mCherry/YFP-3D signal; Fig. 2B, S3 Video). At this time, 16% of YFP^+^ cells contained mCherry-*Pc* fragments (Fig. 2C). We also observed several mCherry-*Pc* iRBCs trapped by YFP^+^ cells without visible signs of internalization (Fig. 2A, S4 Video). Thus, a substantial proportion of subcapsular RP YFP^+^ cells trapped or internalized iRBCs soon after *Pc* infection. These cells were not activated, as indicated by small YFP^+^ cell volume and sphericity (Fig. 2D).

**Figure 3:**
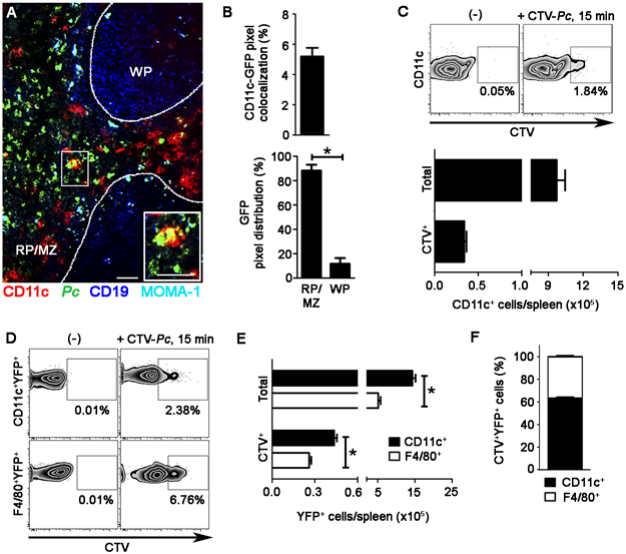
*Ex vivo* analysis of iRBC uptake by splenic DCs soon after *Pc* infection. **(A-C)** B6 mice were i.v. infected with 1 × 10^8^ purified mature CTV-*Pc* iRBCs (flow cytometry) or GFP-*Pc* iRBCs (immunofluorescence). Spleens were analyzed after 15 min and in non-infected controls. **(A)** A representative immunofluorescence image (10x magnification) shows the spleen of an infected B6 mouse. The staining of MOMA-1^+^ metallophilic macrophages and CD19^+^ B cells delineates the RP/MZ from the white pulp (WP). The lower panel details a merged GFP^+^CD11c^+^ cell in the RP. **(B)** Percentage of CD11c pixels colocalized with GFP pixels and percentages of GFP pixel distribution in the splenic RP/MZ and WP were obtained from immunofluorescence images (means ± SD). **(C)** Representative contour plots obtained by flow cytometry show CTV staining in the CD11c^+^ cells of naïve mice (-) and recently infected mice. CD11c^+^ cells in the CD3^-^CD19^-^DX5^-^Ter119^-^ cell population were analyzed, while excluding T cells, B cells, NK cells and RBCs. Data in contour plots show the percentages of CTV^+^ cells in the CD11c^+^ cell population. The numbers of total and CTV^+^CD11c^+^ cells per spleen in recently infected mice were calculated from the data obtained in contour plots (means ± SD). **(D-F)** B6.CD11c-YFP mice were i.v. infected with 1 × 10^8^ purified mature CTV-*Pc* iRBCs. Spleens were analyzed by flow cytometry after 15 min and in non-infected controls. **(D)** Representative contour plots show CTV staining in the CD11c^+^YFP^+^ and F4/80^+^YFP^+^ cells. Data show the percentages of CTV^+^ cells in each population. **(E)** The numbers of total and CTV^+^ CD11c^+^YFP^+^ and F4/80^+^YFP^+^ cells per spleen were calculated from the data obtained in **D** (means ± SD). **(F)** The relative proportions of CD11c^+^ and F4/80^+^ cells in the CTV^+^YFP^+^ cell population were calculated from the data obtained in **E** (means ± SD). In **A**, the scale bars correspond to 50 µm. In **B**, data were calculated using FIJI software. In **B** and **E**, significant differences (p < 0.05) between the indicated groups are designated by *. In **A-F**, one representative experiment out of three (n = 3-4) is shown.

The phagocytic activity of splenic DCs from recently infected B6 mice was also analyzed *ex vivo* by immunofluorescence and flow cytometry. Immunofluorescence revealed approximately 5% CD11c pixels that were colocalized with GFP pixels in those spleens (Fig. 3A and 3B). The majority of GFP-*Pc* iRBCs were trapped inside the RP and MZ (Fig. 3B). Nearly 2% of CD11c^+^ cells internalized Cell Tracer Violet (CTV)-*Pc* parasites (4 × 10^4^ CTV^+^CD11c^+^ cells/spleen), as revealed by flow cytometry (Fig. 3C). Comparable data were obtained with Green Fluorescent Protein (GFP)-*Pc* iRBCs (S1 Table). This phagocytic activity was not restricted to a DC subtype, as subsets of CD11c^+^ cells co-expressing CD11b, CD8, B220 or CD4 were CTV^+^ (S4A Fig.). Considering the numbers of cells per spleen, CD11b^+^CD11c^+^ cells were responsible for most of the parasite clearance carried out by CD11c^+^ cells in recently infected mice (S4B Fig.).

Although 61% of YFP^+^ cells in recently infected B6.CD11c-YFP mice had a DC phenotype, expressing CD11c and MHC class II (I-A) but not F4/80, 20% displayed the phenotype of F4/80^+^ RP macrophages (S5 Fig.). Therefore, we also analyzed the phagocytic activity of the YFP^+^ cell subsets by CIVM and flow cytometry. With injection of a fluorescent anti-F4/80 mAb into mice, CIVM revealed that 17% of cells in the subcapsular RP YFP^+^ cell population were F4/80^+^ soon after infection (Fig. 2E and 2F, S5 Video). Approximately 15% of F4/80^+^YFP^+^ and F4/80^-^YFP^+^ cells internalized Cell Tracker Red CMTPX (CMTPX)-*Pc* parasites (Fig. 2G), but only 20% of the CMTPX^+^YFP^+^ cells were F4/80^+^ (Fig. 2H). Flow cytometry analysis of the YFP^+^ cell subsets showed that a proportion of CD11c^+^ and F4/80^+^ cells was CTV^+^ in B6.CD11c-YFP mice that were recently infected with CTV-*Pc* iRBCs (Fig. 3D). The CD11c^+^ cells made up 63% of the CTV^+^YFP^+^ cell population (4.5 × 10^4^ CTV^+^CD11c^+^YFP^+^ cells/spleen), while 37% of CTV^+^YFP^+^ cells expressed F4/80 (2.5 × 10^4^ CTV^+^F4/80^+^YFP^+^ cells/spleen) (Fig. 3E and 3F).

### Splenic DCs interact with CD4^+^ T cells in CD4^+^ T cell-rich areas and the RP during early *Pc* malaria

**Figure 4:**
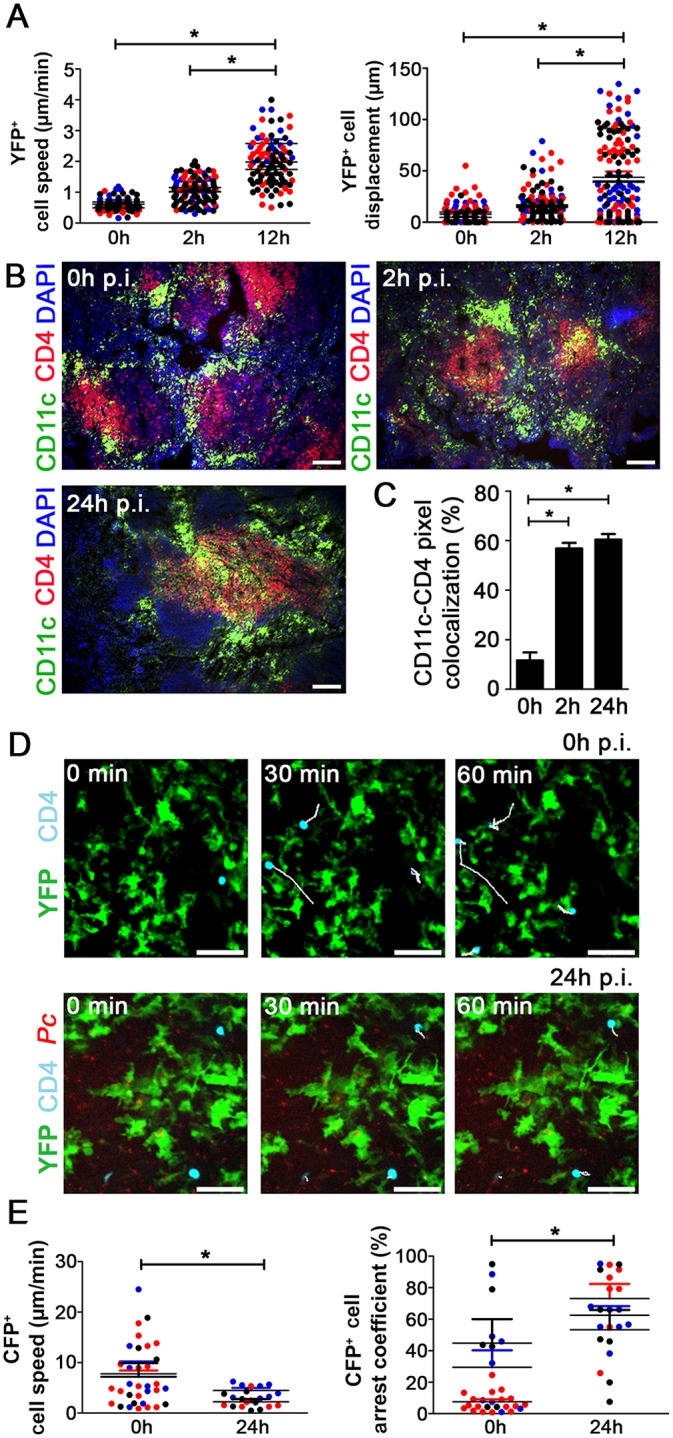
Analysis of the interactions between splenic DCs and CD4^+^ T cells after *Pc* infection. **(A)** B6.CD11c-YFP mice were i.p. infected with 1 × 10^6^ mCherry-*Pc* iRBCs. Spleens were analyzed by CIVM after 2 h and 12 h and in non-infected controls. Speed and displacement of YFP^+^ cells are shown. Black, red and blue dots are from three different experiments. Horizontal lines represent mean values and SEM. **(B** and **C)** B6 mice were i.p. infected with 1 × 10^6^ mCherry-*Pc* iRBCs. Spleens were analyzed after 2 h or 24 h and in non-infected controls. **(B)** Representative immunofluorescence images (10x magnification) show CD11c^+^ cells and CD4^+^ cells in DAPI-stained tissue sections. **(C)** Percentages of CD11c pixels colocalized with GFP pixels in the immunofluorescence images are shown (means ± SD). **(D** and **E)** B6.CD11c-YFP mice were adoptively transferred with 5 × 10^6^ CFP^+^CD4^+^ T cells and i.p. infected with 1 × 10^6^ mCherry-*Pc* iRBCs. Spleens were analyzed by CIVM after 24 h and in non-infected controls. **(D)** Snapshots show the subcapsular RP with CFP^+^CD4^+^ cell tracking. **(E)** Speed and arrest coefficients of CFP^+^CD4^+^ cells are shown. Black, red and blue dots are from three different experiments. Horizontal lines represent mean values and SEM. In **B** and **D**, the scale bars correspond to 100 and 50 µm, respectively. In **A**, **C** and **E**, significant differences (p < 0.05) between the indicated groups are designated by *. In **A** and **E**, data were calculated using Imaris software. Data from three experiments (n = 2) are shown. In **C**, data were calculated from eight images (two images per mouse) using FIJI software. In **B** and **C**, one representative experiment out of three (n = 4) is shown. In **D**, one representative experiment out of three (n = 2) is shown.

Next, we evaluated the dynamics of splenic DCs during early *Pc* malaria. At 12 h p.i., the subcapsular RP YFP^+^ cells from B6.CD11c-YFP mice displayed higher speed and displacement (Fig. 4A). This enhanced motility of YFP^+^ cells correlated with their migration towards CD4^+^ T cell-rich areas. This was evident in immunofluorescences, at 2 h and 24 h p.i., by the presence of yellow areas of merged FITC/PE signal (Fig. 4B) and higher percentages of CD11c-CD4 pixel colocalization (Fig. 4C). We also adoptively transferred CD4^+^ T cells expressing Cyan Fluorescent Protein (CFP) into B6.CD11c-YFP mice to evaluate the interaction of subcapsular RP DCs with CD4^+^ T cells during early *Pc* malaria. In naïve mice, most CFP^+^CD4^+^ cells made transient contacts with YFP^+^ cells (Fig. 4D, S6 Video), and CFP^+^CD4^+^ cells were actively moving inside spleen (Fig. 4E). At 24 h p.i., CFP^+^CD4^+^ cells contacted YFP^+^ cells more stably (Fig. 4D, S7 Video), as indicated by a decrease in CFP^+^CD4^+^ cell speed and an increase in arrest coefficient (Fig. 4E).

### Splenic DCs from the pre-crisis show intense phagocytic activity

**Figure 5:**
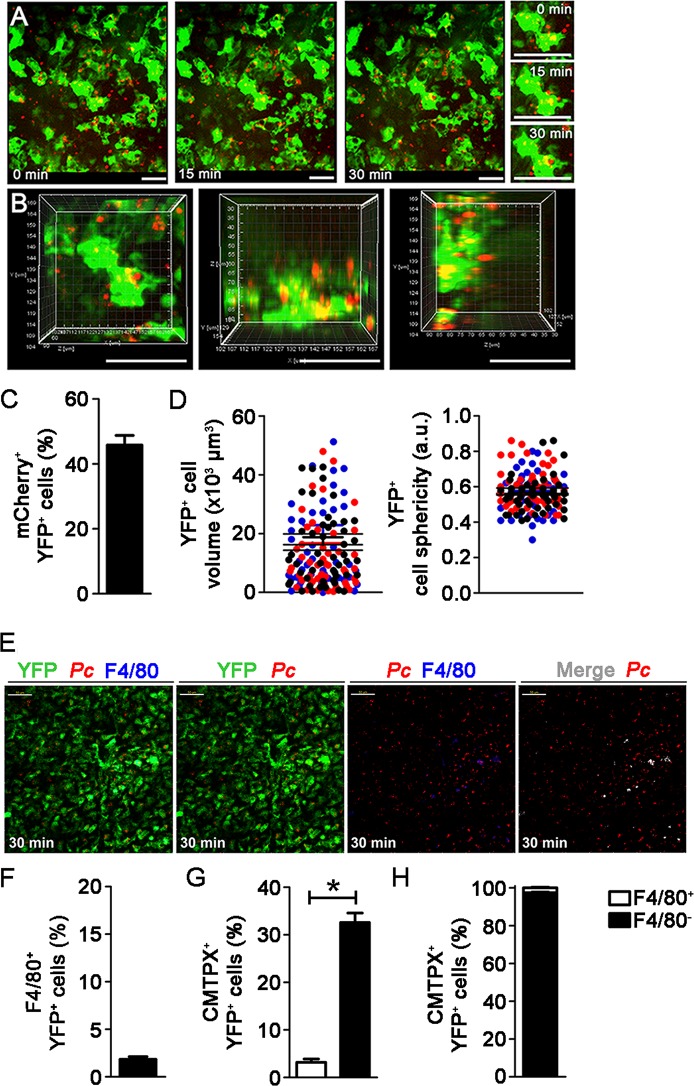
*In vivo* analysis of iRBC uptake by subcapsular RP DCs during pre-crisis. **(A-D)** B6.CD11c-YFP mice were i.p. infected with 1 × 10^6^ mCherry-*Pc* iRBCs. Spleens were analyzed by CIVM after five days, at a time of day when mature parasite stages predominated. **(A)** Serial snapshots show the subcapsular RP and, in the right panel, a detailed image of a YFP^+^ cell (green) upon phagocytosis of an mCherry-*Pc* iRBC (red). **(B)** CIVM 3D animation shows the presence of mCherry-*Pc* iRBC remnants (yellow spots of merged mCherry/YFP-3D signal) inside the YFP^+^ cells. **(C)** Percentage of mCherry^+^ cells in the YFP^+^ cell population is shown (mean ± SEM). **(D)** YFP^+^ cell volume and sphericity are shown. Black, red and blue dots are from three different experiments. Horizontal lines represent mean values and SEM. **(E**-**H)** B6.CD11c-YFP mice were i.p. infected with 1 × 10^6^ *Pc* iRBCs. At five days p.i., mice were i.v. infected with 1 × 10^8^ mature CMTPX-*Pc* iRBCs. After 15 min, mice were injected i.v. with a fluorescent anti-F4/80 mAb and the spleens were analyzed by CIVM. **(E)** Snapshots taken 30 min later show YFP^+^ cells (green), iRBCs (red), F4/80^+^ cells (blue) and merged F4/80^+^YFP^+^ cells (white) in the subcapsular RP. **(F)** Percentage of F4/80^+^ cells in the YFP^+^ cell population is shown (mean ± SEM). **(G)** Percentages of CMTPX^+^ cells in the F4/80^+^YFP^+^ and F4/80^-^YFP^+^ cell subsets are shown (means ± SEM). **(H)** The relative proportions of F4/80^+^ and F4/80^-^ cells in the CMTPX^+^YFP^+^ cell population were calculated from the data obtained in **F** and **G** (means ± SEM). In **A, B** and **E**, the scale bars correspond to 50 µm. One representative experiment out of three (n = 2) is shown. Data were calculated using Imaris software. In **C, D, F, G** and **H**, data were calculated using Imaris software. Data from three experiments (n = 2) are shown. In **G**, significant differences (p < 0.05) between the indicated groups are designated by *.

To investigate whether splenic DCs have a direct role in parasite clearance during pre-crisis, we analyzed the interactions between splenic DCs and iRBCs after five days of infection *in vivo* and *ex vivo*. This possibility was suggested by our data showing that, on day 5 p.i., splenic DCs had an enhanced expression of the phagocytic receptor FcγRI (S6A–S6B Fig.). Notably, we visualized many mCherry-*Pc* iRBCs inside the subcapsular RP, and YFP^+^ cells displayed intense phagocytic activity (Fig. 5A, S8 Video). The presence of intense vacuolization in these DCs was also clear, and we observed some YFP^+^ cells (containing iRBC remnants from previous internalization events) phagocytizing mCherry-*Pc* iRBCs (Fig. 5A, S9 Video). CIVM 3D animations confirmed the internalization of mCherry-*Pc* parasites by YFP^+^ cells (Fig. 5B, S10 Video). This phenomenon was observed in 45% of the YFP^+^ cells (Fig. 5C). At five days p.i., YFP^+^ cells were activated and displayed higher cell volume and lower cell sphericity than those from recently infected mice (Fig. 5D; S1 Table). On day 5 p.i., the CD11c^+^ cells also expressed higher levels of MHC class II, CD80 and CD86 compared to those from naïve mice (S6C–S6D Fig.).

**Figure 6:**
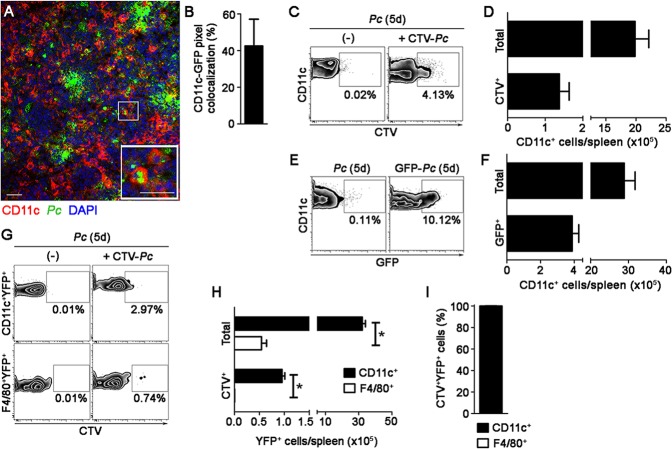
*Ex vivo* analysis of iRBC uptake by splenic DCs during pre-crisis. **(A** and **B)** B6 mice were i.p. infected with 1 × 10^6^ GFP-*Pc* iRBCs. Spleens were analyzed after five days, at a time of day when mature parasite stages predominated. **(A)** A representative immunofluorescence image (10x magnification) represents the spleens of GFP-*Pc*-infected mice. The lower panel details a merged GFP^+^CD11c^+^ cell. **(B)** Percentage of CD11c pixels colocalized with GFP pixels in the immunofluorescence images is shown (mean ± SD). **(C** and **D)** B6 mice were i.p. infected with 1 × 10^6^ *Pc* iRBCs. At five days p.i., half of the B6 mice were i.v. re-infected with 1 × 10^8^ mature CTV-*Pc* iRBCs. Spleens were analyzed by flow cytometry after 15 min. **(C)** Representative contour plots show CTV staining in the CD11c^+^ cells of *Pc*-infected mice that were re-infected or not with CTV-*Pc* iRBCs. CD11c^+^ cells in the CD3^-^CD19^-^DX5^-^Ter119^-^ cell population were analyzed. Data show the percentages of CTV^+^ cells in the CD11c^+^ cell population. **(D)** Numbers of total and CTV^+^CD11c^+^ cells per spleen in re-infected mice were calculated from the data obtained in **C** (means ± SD). **(E** and **F)** B6 mice were i.p. infected with 1 × 10^6^ *Pc* iRBCs or GFP-*Pc* iRBCs. Spleens were analyzed after five days, at a time of day when mature parasite stages predominated. **(E)** Representative contour plots obtained by flow cytometry show GFP staining in the CD11c^+^ cells of mice that were infected with *Pc* iRBCs or GFP-*Pc* iRBCs. CD11c^+^ cells in the CD3^-^CD19^-^DX5^-^Ter119^-^ cell population were analyzed. Data show the percentages of GFP^+^ cells in the CD11c^+^ cell population. **(F)** Numbers of total and GFP^+^CD11c^+^ cells per spleen in GFP-*Pc*-infected mice were calculated from the data obtained in **E**. **(G**-**I)** B6.CD11c-YFP mice were i.p. infected with 1 × 10^6^ *Pc* iRBCs. At five days p.i., half of the B6.CD11c-YFP mice were i.v. re-infected with 1 × 10^8^ mature CTV-*Pc* iRBCs. Spleens were analyzed by flow cytometry after 15 min. **(G)** Representative contours plots show CTV staining in the CD11c^+^YFP^+^ and F4/80^+^YFP^+^ cells. Data show the percentages of CTV^+^ cells in each population. **(H)** The numbers of total and CTV^+^ CD11c^+^YFP^+^ and F4/80^+^YFP^+^ cells per spleen were calculated from the data obtained in **G** (means ± SD). **(I)** The relative proportions of CD11c^+^ and F4/80^+^ cells in the CTV^+^YFP^+^ cell population were calculated from the data obtained in **H** (means ± SD). In **A**, the scale bars correspond to 50 µm. In **B**, data were calculated using FIJI software. In**H**, significant differences (p < 0.05) between the indicated groups are designated by *. In **A-I**, one representative experiment out of three (n = 3-4) is shown.

Immunofluorescence corroborated the significant role of splenic DCs in the widespread iRBC phagocytosis observed during pre-crisis. The percentages of CD11c pixels that colocalized with GFP pixels reached up to 40% in spleens from B6 mice on day 5 p.i. (Fig. 6A and 6B). Flow cytometry confirmed that splenic DCs were able to phagocytize iRBCs during pre-crisis. When mature CTV-*Pc* iRBCs were i.v. injected into B6 mice on day 5 p.i., approximately 4% of splenic DCs were CTV^+^ (1.4 × 10^5^ CTV^+^CD11c^+^ cells/spleen) (Fig. 6C and 6D). Phagocytic activity was not restricted to a particular DC subtype, as a proportion of all subsets studied internalized iRBCs during pre-crisis (S4A Fig.). However, CTV^+^ CD11b^+^CD11c^+^ and CD8^+^CD11c^+^ cell numbers were significantly higher per spleen than those of other DC subsets (S4B Fig.). In addition, on day 5 p.i., 10% of CD11c^+^ cells from mice infected with GFP-*Pc* iRBCs were GFP^+^ (4 × 10^5^ CTV^+^CD11c^+^ cells/spleen) (Fig. 6E and 6F). Comparatively, we observed substantially higher activation and phagocytic activity both *in vivo* and *ex vivo* in the splenic DCs during pre-crisis (S1 Table). Furthermore, a significantly higher frequency of iRBC uptake was detected using CIVM in comparison with flow cytometry.

Notably, flow cytometry analysis of splenic YFP^+^ cells from B6.CD11c-YFP mice during pre-crisis showed a sharp reduction in the percentages of F4/80^+^ cells so that the great majority of the YFP^+^ cell population presented with a classical DC phenotype (S5 Fig.). Moreover, a large fraction of CD11c^+^YFP^+^ cells in these mice expressed higher levels of MHC class II molecules in comparison to those in recently infected B6.CD11c-YFP mice. This observation was confirmed by CIVM, which revealed a reduction of F4/80^+^YFP^+^ cells in the subcapsular RP of B6.CD11c-YFP mice on day 5 p.i. (Fig. 5E and 5F, S11 Video). Due to the incremental number of CD11c^+^ cells in the YFP^+^ cell population, almost all of the phagocytic activity of YFP^+^ cells was imputed to DCs during pre-crisis, as observed by CIVM (Fig. 5G and 5H) and by flow cytometry (Fig. 6G, 6H and 6I).

### *Pc* phagocytosis by splenic DCs is no longer observed during crisis

**Figure 7:**
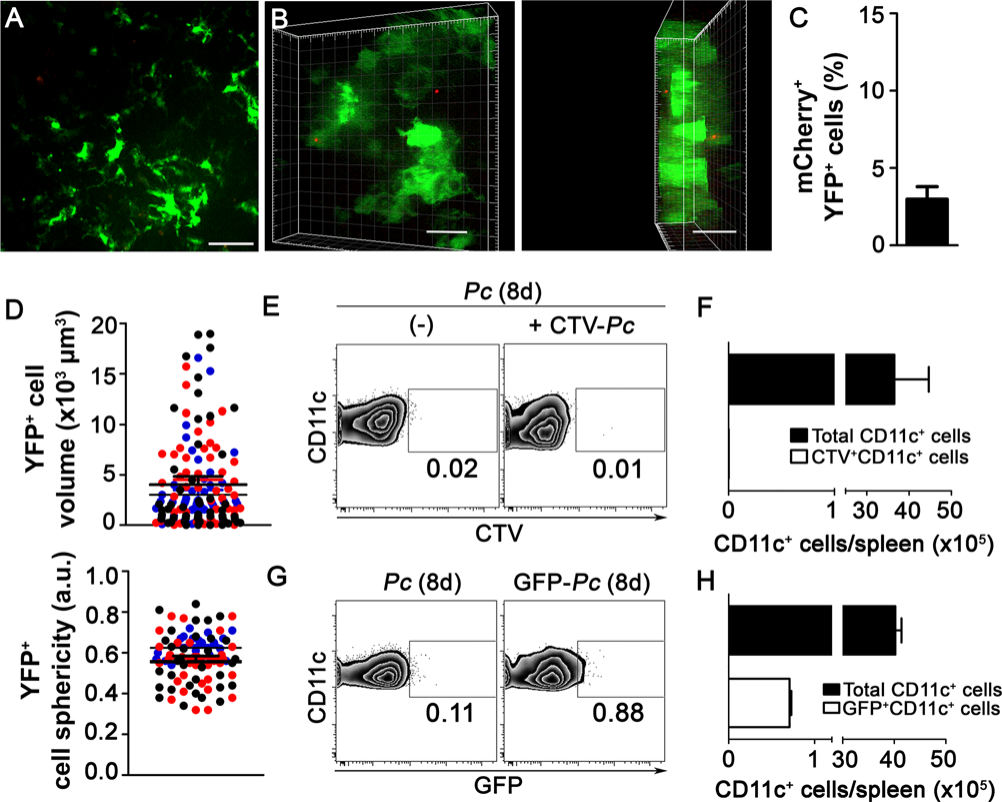
*In vivo* and *ex vivo* analysis of iRBC uptake by splenic DCs during crisis. **(A-D)** B6.CD11-YFP mice were i.p. infected with 1 × 10^6^ mCherry-*Pc* iRBCs. Spleens were analyzed by CIVM after eight days, at a time of day when mature parasite stages predominated. **(A)** A snapshot shows the subcapsular RP. **(B)** CIVM 3D animation shows the presence of few mCherry-*Pc* iRBCs (red) attached to YFP^+^ cells (green). **(C)** Percentage of mCherry^+^ cells in the YFP^+^ cell population is shown (mean ± SEM). **(D)** YFP^+^ cell volume and sphericity are shown. Black, red and blue dots are from three different experiments. Horizontal lines represent mean values and SEM. **(E** and **F)** B6 mice were i.p. infected with 1 × 10^6^ *Pc* iRBCs. At eight days p.i., half of the B6 mice were re-infected i.v. with 1 × 10^8^ purified mature CTV-*Pc* iRBCs. Spleens were analyzed by flow cytometry after 15 min. **(E)** Representative contour plots show CTV staining in the CD11c^+^ cells of *Pc*-infected mice that were re-infected or not with CTV-*Pc* iRBCs. CD11c^+^ cells in the CD3^-^CD19^-^DX5^-^Ter119^-^ cell population were analyzed. Data show the percentages of CTV^+^ cells in the CD11c^+^ cell population. **(F)** The numbers of total and CTV^+^CD11c^+^ cells per spleen were calculated from the data obtained in **E** (means ± SD). **(G** and **H)** B6 mice were i.p. infected with 1 × 10^6^ *Pc* iRBCs or GFP-*Pc* iRBCs. Spleens were analyzed after eight days, at a time of day when mature parasite stages predominated. **(G)** Representative contour plots obtained by flow cytometry show GFP staining in CD11c^+^ cells of mice that were infected with *Pc* iRBCs or GFP-*Pc* iRBC. CD11c^+^ cells in the CD3^-^CD19^-^DX5^-^Ter119^-^ cell population were analyzed. Data show the percentages of GFP^+^ cells in the CD11c^+^ cell population. **(H)** Numbers of total and GFP^+^CD11c^+^ cells per spleen were calculated from the data obtained in **G**. In **A** and **B**, the scale bars correspond to 50 µm and 30 µm, respectively. One representative experiment out of three (n = 2) is shown. In **C** and **D**, data were obtained using Imaris software. Data from three experiments (n = 2) are shown. In **E-H**, one representative experiment out of three (n = 5) is shown.

During the crisis phase of acute *Pc* malaria, profound modifications in the splenic architecture occur, resulting in RP closure [36]. Therefore, we extended our study into this phase of the disease. CIVM revealed only occasional mCherry-*Pc* iRBCs trapped by subcapsular RP YFP^+^ cells in B6.CD11c-YFP mice on day 8 p.i. (Fig. 7A and 7B, S12 Video), and yellow spots of merged mCherry/YFP-3D signal were infrequent (Fig. 7C). At that same time point, YFP^+^ cell volumes were smaller than during pre-crisis (Fig. 7D, S1 Table). YFP^+^ cell sphericity was reduced in mice on days 5 and 8 p.i. compared with naïve mice (Fig. 7D, S1 Table). Flow cytometry also revealed poor phagocytosis by splenic DCs, a process that was investigated both when mice were re-infected i.v. with mature CTV-*Pc* iRBCs and when mice were i.p. infected with GFP-*Pc* iRBCs (Fig. 7E, 7F, 7G and 7H). These data indicate that splenic DCs could be primarily involved in antigen presentation rather than in phagocytosis during crisis, as CD11c^+^ cells expressed high levels of MHC class II and CD80 on day 8 p.i. (S6C–S6D Fig.).

## Discussion

The depletion of phagocytes *in vivo* allowed us to clearly demonstrate the key role of DCs in the protection against experimental blood-stage malaria. Abundant CD11c expression is a well-known marker for DCs, which are primary targets of DTx treatment in B6.CD11c-DTR mice [38]. Nevertheless, MZ macrophages are also depleted in DTx-treated B6.CD11c-DTR mice due to ectopic expression of the DTx receptor transgene [39]. The role of DCs was established in our study by comparing the disease progression in DTx-treated B6.CD11c-DTR mice and in B6 mice treated with a low dose of ClLip, which selectively depletes MZ macrophages within splenic phagocytes [39], [40]. The significant contribution of DCs in the control of *Pc* malaria was suggested by data showing the worsening of the disease in DTx-treated B6.CD11c-DTR mice, while the elimination of MZ macrophages by the ClLip treatment did not alter the course of infection in B6 mice. Our data also showed that splenic DCs are required for CD4^+^ T cell proliferation and IFN-γ production during *Pc* infection. The complete abrogation of these responses in DTx-treated B6.CD11c-DTR mice, but not in ClLip-treated B6 mice, demonstrated that other splenic phagocytes such as MZ and RP macrophages did not replace DCs in the initiation of CD4^+^ T cell responses to *Pc* infection.

Our first evidence suggesting that DCs could directly contribute to parasite clearance was the effect of DC depletion on the increase of parasitemia and the reduction of body weight during the first days of blood-stage *Pc* and *Py* malaria. DCs were also required to control the early parasitemia following infection with *Pb* sporozoites. The early protective role of DCs could not be completely attributed to the need for these cells to activate T cells, which take longer to produce IFN-γ and induce antibody secretion during experimental malaria. The splenocytes obtained four and five days after *Pc* infection still require further stimulation with iRBCs *in vitro* to differentiate into effector cells [41], [42], while the *ex vivo* production of IFN-γ and antibodies coincides with the drop of parasitemia a week after infection [42], [43]. Using *in vivo* and *ex vivo* approaches, we unequivocally demonstrated here that the subcapsular RP DCs recognize and phagocytize mature iRBCs during the first encounter and pre-crisis, while spleen closure coincides with limited *Pc* phagocytosis by DCs during crisis. Although the splenic DCs are thought to be a major DC population in intimate contact with the bloodstream, these cells may act together with other DCs outside the spleen to clear *Plasmodium* parasites. This idea is supported by studies in splenectomized mice showing that other reticuloendothelial organs, such as the liver, effectively substitute for the phagocytic functions of the spleen in protecting against *Pc* malaria [22], [44]. In fact, hepatic CD11c^+^ DCs are also capable of internalizing iRBCs in the liver sinusoids during acute *Pc* infection [45].

CIVM allowed us to visualize the interaction between subcapsular RP DCs and iRBCs in great detail. In naïve mice, these cells actively extended protrusions and dendrites, as previously shown [26]. Soon after infection, we observed iRBCs being trapped by DCs that had a non-activated phenotype. The majority of these cells showed a classical DC phenotype, but a proportion of them exhibited strong labeling for F4/80, a marker of RP macrophages that is also expressed by a subset of DCs in the skin [46]. Another study reporting a similar observation concluded that, based on their dendritic morphology, subcapsular RP F4/80^+^YFP^+^ cells represent a subset of peripheral tissue DCs [26]. Although we did not visualize phagocytosis of iRBCs in recently infected mice, the detection of *Pc* remnants inside subcapsular RP DCs suggests that iRBC uptake had occurred. In fact, parasite antigen presentation is likely to occur soon after *Pc* infection, similar to the process observed during *L. monocytogenes* infection [47]. During the first day p.i., subcapsular RP DCs displayed high motility and made stable contacts with CD4^+^ T cells. DCs also migrated rapidly to T cell-rich areas following *Pc* infection, a process that might involve chemokine signaling as suggested by studies in CCR7-knockout mice [48].

Here, for the first time, we observed the phagocytosis of iRBCs during pre-crisis *in vivo*. This occurred in a large number of subcapsular RP DCs, such that up to half of this population presented with *Pc* remnants. The great majority of these cells had a classical DC phenotype, which was characterized by negative staining for F4/80 and high expression of both MHC class II and costimulatory molecules. It is notable that these cells displayed an activated phenotype. Even if most subcapsular RP DCs during pre-crisis are immature cells that recently migrated to the spleen [49], it is expected that DC activation leads to their maturation and consequent blockade of phagocytic activity, allowing the cellular machinery to be restructured for antigen presentation [28]. In agreement with our data, a previous report determined that the peak of *in vitro* iRBC uptake by splenic DCs occurred at five days p.i., in parallel with the increase in the expression of MHC class II and costimulatory molecules [34]. In both studies, the phagocytic activity was not restricted to a particular DC subset. Our *ex vivo* data implicate CD11b^+^ and CD8^+^ DCs in most of the parasite clearance imputed to splenic DCs in mice both soon after infection and at the pre-crisis phase. Consistent with the immune response to acute *Pc* malaria, the CD11b^+^ and CD8^+^ DC subsets are known to be specifically involved in antigen presentation to CD4^+^ T cells and IL-12 production, respectively [50], [51]. Furthermore, both subsets of DCs are able to induce IFN-γ production by parasite-specific T cells during *Pc* infection [29]. Another important observation during pre-crisis was a sharp decline in the population of F4/80^+^YFP^+^ cells, a phenomenon that also occurred to splenic F4/80^+^ macrophages after the parasitemia peak (unpublished data). Because DCs have a higher turnover than F4/80^+^ macrophages [47], a possible explanation for our results is that a proportion of these phagocytes died after ingesting *Pc* parasites and only DCs were rapidly replaced. This process would substitute F4/80^+^ macrophages, a resident RP population that is primarily required to maintain tissue homeostasis [52], to inflammatory phagocytes. An alternative explanation is the down-regulation of the F4/80 molecule due to macrophage activation as reported during mycobacterial infection [53]. The F4/80^+^YFP^+^ cells could also have migrated to other locations such as the splenic T cell-rich areas.

During crisis, the down-regulation of the phagocytic function of splenic DCs coincided with the period of spleen closure. This was demonstrated here by *in vivo* images showing a few iRBCs in the subcapsular RP at eight days p.i., when parasitemias were even higher than at five days p.i.. The decline in iRBC uptake was also associated with the maximum expression of MHC class II and CD80 molecules by splenic DCs, which indicates that complete DC maturation was only achieved during crisis. This idea is corroborated by a previous study that reported a decrease to baseline levels of the *in vitro* uptake of the iRBCs by splenic DCs at day 8 p.i. [34]. Thus, in addition to spleen closure and the subsequent blockade of iRBC entry inside the RP, splenic DCs seem to lose the ability to phagocytize parasites, while concomitantly increasing their ability to present cognate antigens. This is an interesting observation because, during crisis, most of the lymphocytes that are activated during early *Pc* infection undergo apoptosis [54], [55]. Thus, it is possible that mature DCs are required to expand and differentiate the few remaining T cells, giving rise to the memory response to malaria [56], [57].

The quantification of iRBC phagocytosis *ex vivo* by flow cytometry yielded substantially lower percentages of *Pc* ^+^ DCs compared with *in vivo* data obtained by CIVM. This discrepancy may result from differences in the fluorescence detection thresholds of CIVM and flow cytometry, the DC subpopulations examined by these techniques (subcapsular RP DCs or total splenic DCs, respectively) or the fluorochrome labeling of the iRBCs (mCherry, GFP, CTV or CMTPX). Another possible explanation for the low detection of iRBC uptake by flow cytometry is the rapid iRBC degradation or fluorochrome quenching [8], such that *Pc* remnants were only identified inside DCs shortly after phagocytosis. Previously, low frequencies of iRBC uptake were also detected by flow cytometry in migrating monocytes [8], [27]. Immunofluorescence confirmed that splenic DCs, particularly those localized inside the RP and MZ, play a major role in the clearance of iRBCs during acute *Pc* infection. Although this technique did not efficiently discriminate single cells, the percentages of CD11c-GFP pixel co-localization were comparable to those of *Pc* ^+^ DCs obtained by CIVM.

The *in vivo* approaches used in this study indicate that, beyond the classical role of DCs in antigen presentation, these cells also contribute to the direct elimination of iRBCs during acute *Plasmodium* infection. For several days after *Pc* infection, subcapsular RP DCs were highly efficient in the recognition and capture of iRBCs. Complete DC maturation appeared to be achieved only during crisis when restructuring of the spleen might facilitate the development of the acquired immunity. Taking into account the specifics of different parasite-host interactions, we speculate whether our findings in mouse models could be applied to human malaria. The adhesion of *P. falciparum* iRBCs to human monocyte-derived DCs through the scavenger receptor CD36 has been shown to inhibit DC maturation and subsequently reduce their capacity to activate T cells [58]. This observation was interpreted as the impairment of the DC function during *P. falciparum* infection. However, our data showing the induction of FcγRI in splenic DCs during pre-crisis open the possibility that recognition of opsonized iRBCs through this receptor can overcome the down-regulatory activity of CD36 signaling. Thus, the opposite effects of malaria on DC function could be related to the different activation profiles of DCs, which are greatly influenced by the surrounding tissue microenvironment, rather than other factors previously discussed such as different species and strains of hosts and parasites [59]. Together, our data add novel information to this area of immunology and demonstrate that *in vivo* imaging may help to unravel the mechanisms underlying protective immunity against malaria.

## Materials and Methods

### Mice, parasites and infections

Six- to eight-week-old B6, B6.CD11c-DTR [28], B6.CFP [60] and B6.CD11c-YFP mice [61] were bred under specific pathogen-free conditions at the Animal Facilities of Instituto Gulbenkian de Ciência (IGC), Instituto de Ciências Biomédicas at the Universidade de São Paulo (ICB-USP) or Institut de Transgénose Orléans-Villejuif. *Pc* (AS strain), *Py* (XL strain) and mCherry-*Pc* were maintained previously as described [62], [63]. GFP-*Pc* parasites were selected by treatment with pyrimethamine (Sigma-Aldrich, USA) [64]. The Instituto de Medicina Molecular at the Universidade de Lisboa provided *Anopheles stephensi* mosquitoes infected with *Pb* (ANKA strain). Mice were infected intraperitoneally (i.p.) with 1 × 10^6^ iRBCs (blood from infected mice), and intravenously (i.v.) with 1 × 10^8^ iRBCs or 1 × 10^3^ sporozoites. Purified iRBCs were used where specified. The iRBCs were obtained during a period of the circadian cycle in which mature stages predominated (>95% late trophozoites/schizonts).

### Ethics statement

All procedures were in accordance with the national regulations of Conselho Nacional de Saúde and Colégio Brasileiro em Experimentação Animal (COBEA) and Federation of European Laboratory Animal Science Associations (FELASA). The protocols were approved by the Comissão de Ética no Uso de Animais (CEUA) of ICB-USP, São Paulo, Brazil under permit numbers 0036/2007 and 0174/2011, and by FELASA under permit number AO10/2010.

### DTx and ClLip treatments

To deplete CD11c^+^ cells, B6.CD11c-DTR mice were injected i.p. with a single dose of 2 ng/g body weight of DTx (Sigma-Aldrich) 24 h before iRBC infection or 48 h after sporozoite infection. This dose is half of the one previously established to deplete CD11c^+^ cells [65] and it was used to reduce drug toxicity. To deplete MARCO^+^/MOMA1^+^ cells, B6 mice were injected i.v. with 8.5 µg/g body weight of ClLip 24 h before infection [40]. Phosphate buffered saline (PBS) or PBS liposomes (PBSLip) were injected as controls. The procedures to obtain ClLip and PBSLip were described elsewhere [66].

### CTV or CMTPX staining of purified iRBCs

Blood from infected B6 mice was resuspended in 1 ml PBS, pipetted over 5 ml of 74% Percoll (GE Healthcare, USA) and centrifuged (2500 x g, acceleration/break 5/0) for 30 min at room temperature (RT). The top cell layers were collected and washed with complete RPMI 1640 medium (supplemented with 10% heat-inactivated fetal calf serum, 100 U/ml penicillin, 100 µg/ml streptomycin, 50 µM 2-mercaptoethanol, 2 mM L-glutamine and 1 mM sodium pyruvate; Life Technologies, USA). Purified iRBCs (>95% purity) were stained with CTV or CMTPX, following the manufacturer’s instructions (Life Technologies).

### CIVM analysis

B6.CD11c-YFP mice infected with mCherry-*Pc* iRBCs were deeply anesthetized i.p. with 55 ng/g body weight of ketamine (Imalgene 1000, Merial, USA) and 0.85 ng/g body weight of xylazine (Rompun 2%, Bayer, Germany). Spleens were externalized by a 1 cm incision just below the ribcage. Mice were placed above a metal plate with a coverslip and immobilized without disrupting the vasculature or splenic connective tissue. Live imaging was carried out with an Eclipse Ti microscope (Nikon Instruments Inc., Japan) equipped with an Andor Revolution XD system (Andor Technology, UK), a Yokogawa CSU-X1 spinning disk unit (Andor Technology), a 20x PLAN APO VC objective (Nikon Instruments Inc.) and a 1.5x auxiliary magnification system (Nikon Instruments Inc.). Data were processed with MicroManager 1.2 (General Public License, NIH, USA). For each movie, 28 µm Z-sections with 4 µm Z-steps were acquired for 30 min. Imaris X64 7.0.0. (Andor Technology) was used to edit images and to determine the percentage of mCherry^+^YFP^+^ cells, as well as the CD11c^+^ cell volume and sphericity. In other cases, B6.CD11c-YFP mice were adoptively transferred with 5 × 10^6^ splenic CD4^+^ T cells from B6.CFP mice (purified by FACS sorting using a FACSAria device; BD Biosciences). These mice were infected as described above and processed 24 h later. Imaris was used to edit images and to determine CD11c^+^ cell speed and displacement, as well as the coefficients of CFP^+^CD4^+^ T cell speed and arrest.

B6.CD11c-YFP mice infected with CMTPX-*Pc* iRBCs were injected i.v. with PE-conjugated anti-F4/80 mAbs (200 ng/g body weight) and deeply anesthetized to externalize the spleen as described above. Live imaging was carried out with a Zeiss LSM 780-NLO confocal microscope (Zeiss, Germany). Data were processed with Zen 2012 software (Zeiss, Germany). In each movie, 28 µm Z-sections with 2 µm Z-steps were acquired for 30 min. Imaris was used to edit images and to determine the percentages of CMTPX^+^ cells.

### Flow cytometry analysis

Mice were sacrificed and PBS-perfused to remove circulating iRBCs. Spleens were harvested, and the remaining RBCs were lysed with ACK lysis buffer. Splenocytes (1 × 10^6^) were stained with fluorescent monoclonal antibodies (mAbs) against CD3, CD4, CD11c, CD69, CD11b, CD80, CD86, I-A^b^, B220, CD36, CD64 (FcγRI), DX5 and Ter119 (BD Biosciences, USA), F4/80 (eBiosciences, USA), and MOMA-1 and MARCO (Abcam, UK). Cells were analyzed by flow cytometry (FACSCanto; BD Biosciences) with FlowJo 9.5.3. (Tree Star Inc., USA).

### Analysis of CD4^+^ T cell proliferation and IFN-γ production

Splenocytes (3 × 10^7^) were resuspended in 1 ml PBS with 0.1% BSA (bovine serum albumin; Sigma-Aldrich) and stained with CFSE (carboxyfluorescein succinimidyl ester; Life Technologies) at a final concentration of 5 μM for 20 min at 37°C. Cells (1 × 10^6^) were cultured in complete RPMI 1640 medium for 72 h at 37°C with 5% CO_2_ in the presence of iRBCs (3 × 10^6^). Cells were then stained with fluorescent mAbs against CD3 and CD4, and proliferation was assessed by flow cytometry. IFN-γ was quantified in the supernatants using the OptEIA IFN-γ kit (BD Biosciences).

### Immunofluorescence analysis

GFP-*Pc* iRBC-infected B6 mice were sacrificed and PBS-perfused. Spleens were removed and frozen in Tissue-Tek OCT (Sakura Fineteck, Japan). Sections 8 µm thick were cut with a CM3050S Cryostat (Leica, USA) and fixed with 1% paraformaldehyde (Alfa Aesar, USA) for 30 min at RT. Sections were incubated with anti-CD16/CD32 mAb (Fc block; BD Biosciences) for 30 min followed by incubation in a humidified dark chamber with fluorescent mAbs against CD11c, CD19, CD3, CD4 (BD Biosciences) and MOMA-1 (Abcam) for 2 h at RT. Sections were then stained for 5 min with 0.5 μg/ml DAPI (4',6-diamidino-2-phenylindole; Sigma-Aldrich), washed with PBS and mounted with Fluoromount-G (Southern Biotechnologies, USA). Images were acquired with a DMRA2 fluorescence microscope (Leica) and MetaMorph software (Molecular Devices Inc., USA). Image analysis was performed with Photoshop CS4 (Adobe Inc., USA). Percentages of CD11c-GFP/CD11c-CD4 pixel colocalization and of GFP pixel distribution in the spleen were calculated using FIJI for Windows 64-bit (*Colocalization threshold* and *Mixture Modeling Thresholding* plugins, respectively; General Public License, NIH, USA).

### Statistical analysis

Results were analyzed with Prism 5 software (Graph Pad) using ANOVA or Student’s t-tests. The existence of a normal distribution was confirmed using the Kolmogorov-Smirnov test. Differences were considered statistically significant at *p* < 0.05.

## Supporting Information

S1 Fig Effects of DTx treatment on splenic DCs and MZ macrophages. **(A** and **B)** B6 and B6.CD11c-DTR mice were treated with DTx or PBS, and their spleens were analyzed by flow cytometry after 24 h. **(A)** Representative contour plots show the depletion of CD11c^+^ and MARCO/MOMA-1^+^(CD11b^+^) cells, but not of F4/80^+^ cells, in DTx-treated B6.CD11c-DTR mice. Data show the percentages of CD11c^+^, F4/80^+^ and MARCO/MOMA-1^+^ cells in the splenocyte population. **(B)** The numbers of CD11c^+^, F4/80^+^ and MARCO/MOMA-1^+^ cells per spleen are shown. In **B**, significant differences (p < 0.05) between all other groups are designated by *. In **A** and **B**, one representative experiment out of three (n = 3) is shown.
(PDF)

S2 Fig Effects of MZ macrophage depletion on acute *Pc* malaria. **(A-D)** B6 mice were treated with either a low dose of ClLip to deplete MARCO^+^ and MOMA-1^+^ macrophages or with PBSLip as controls. The mice were i.p. infected with 1 × 10^6^ *Pc* iRBCs 24 h later. **(A)** Representative contour plots obtained 24 h after treatment by flow cytometry confirm the efficiency of ClLip-induced depletion of MARCO^+^ and MOMA-1^+^ cells without affecting CD11c^+^I-A^+^ and F4/80^+^ cells. Data show the percentages of MARCO^+^, MOMA-1^+^, CD11c^+^I-A^+^ and F4/80^+^ cells in the splenocyte population. **(B)** Parasitemia curves are shown (means ± SD). **(C)** Survival curves are shown. **(D)** Data show the percentages of proliferating CFSE^low^CD4^+^ T cells and IFN-γ concentrations in the supernatants of spleen cell cultures stimulated for 72 h with iRBCs (means ± SD). In **A-D**, one representative experiment out of three (n = 5) is shown.
(PDF)

S3 Fig Effects of DC depletion on the blood stages of infection with *Py* iRBCs or *Pb* sporozoites. **(A-C)** B6 and B6.CD11c-DTR mice were treated with either DTx to deplete CD11c^+^ cells or PBS as a control. The mice were i.p. infected with 1 × 10^6^ *Py* iRBCs 24 h later. **(A)** Parasitemia curves are shown (means ± SD). **(B)** Variations in body weight relative to day 0 are shown (means ± SD). **(C)** Survival curves are shown. **(D-F)** B6 and B6.CD11c-DTR mice were i.v. infected with 1 × 10^3^ *Pb* sporozoites. After 48 h, the mice were treated with DTx to deplete CD11c^+^ cells at the beginning of blood stage. **(D)** Parasitemia curves are shown (means ± SEM). **(E)** Variations in body weight relative to day 0 are shown (means ± SEM). **(F)** Survival curves are shown. In **A-F**, significant differences (p < 0.05) between the indicated groups are designated by *. In A-C, one representative experiment out of three (n = 3-4) is shown. In D-F, data from three experiments (n = 2-3) are shown.
(PDF)

S4 Fig Phagocytosis of iRBCs by splenic DC subsets throughout acute *Pc* malaria. Spleens were analyzed 15 min after i.v. injection of 1 × 10^8^ mature CTV-*Pc* iRBCs (dark line histograms) or PBS (filled histograms) in B6 mice at zero, five or eight days p.i. with 1 × 10^6^ *Pc* iRBCs. **(A)** Representative histograms obtained by flow cytometry show CTV staining in the splenic DC subsets (CD11b^+^, CD8^+^, B220^+^ or CD4^+^). Data show the percentages of CTV^+^ cells in each subset. **(B)** Numbers of total and CTV^+^CD11c^+^ cells per spleen were calculated from the data obtained in **A**. In **B**, significant differences (p < 0.05) between the DC subsets at different days p.i. are designated by *. In **A** and **B**, one representative experiment out of three (n = 5) is shown.
(PDF)

S5 Fig Phenotypic analysis of YFP^+^ cells soon after *Pc* infection and during pre-crisis. **(A** and **B)** Spleens were analyzed 15 min after i.v. injection of 1 × 10^8^ mature CTV-*Pc* iRBCs in B6.CD11c-YFP mice at zero or five days p.i. with 1 × 10^6^ *Pc* iRBCs. **(A)** Representative contour plots show the gate strategy for analysis of YFP^+^ cells in naïve mice. Data show the percentages of singlets, leukocytes and YFP^+^ cells in each contour plot. **(B)** Representative contour plots show CD11c and F4/80 staining in the YFP^+^ cells. Data show the percentages of these cells in the YFP^+^ cell population. Histograms show MHC class II (I-A) staining in CD11c^+^YFP^+^ cells. The fluorescence minus one (FMO) control was obtained in CD11c^+^YFP^+^ cells from a [*Pc* (0) + CTV-*Pc*] mouse (filled histogram). In **A** and **B**, one representative experiment out of three (n = 3) is shown.
(PDF)

S6 Fig Expression of activation markers in splenic DCs throughout acute *Pc* malaria. B6 mice were i.p. infected with 1 × 10^6^ *Pc* iRBCs. At zero, five or eight days p.i., spleens were analyzed by flow cytometry. **(A)** Representative histograms show the expression of CD36 and FcγRI in CD11c^+^ cells. The corresponding FMO control for each marker is represented by the filled histograms. **(B)** Median fluorescence intensity (MFI) was calculated from the data obtained in **A** (means ± SD). **(C)** Representative histograms show the expression of MHC class II (I-A), CD80 and CD86 molecules in CD11c^+^ cells. The corresponding FMO control for each marker is represented by the filled histograms. **(D)** MFI was calculated from the data obtained in **C** (means ± SD). In **B** and **D**, significant differences (p < 0.05) between the indicated groups are designated by *. In **A-D**, one representative experiment out of three (n = 5) is shown.
(PDF)

S1 Table Comparative analyses of *in vivo* and *ex vivo* approaches to the study of splenic DCs throughout acute *Pc* malaria. The data compare several parameters in recently infected mice and mice on days 5 p.i. (pre-crisis) and 8 p.i. (crisis). For *in vivo* analyses, the following two experiments were performed: 1) B6.CD11c-YFP mice were i.v. infected with 1 × 10^8^ mature mCherry-*Pc* iRBCs, and spleens were evaluated 15 min later. 2) B6.CD11c-YFP mice were i.p. infected with 1 × 10^6^ mCherry-*Pc* iRBCs, and spleens were analyzed at five or eight days p.i., at a time of day when mature parasite stages predominated. For *ex vivo* analyses, the following two experiments were performed: 1) B6 mice were i.v. infected with 1 × 10^8^ purified mature CTV-*Pc* iRBCs (flow cytometry) or GFP-*Pc* iRBCs (flow cytometry and immunofluorescence), and spleens were evaluated 15 min later. 2) B6 mice were i.p. infected with 1 × 10^6^ GFP-*Pc* iRBCs (flow cytometry and immunofluorescence), and spleens were evaluated after five or eight days p.i., at a time of day when mature parasite stages predominated. Data were compiled from figs. 2, 3, 5 and 6. Significant differences (p < 0.05) between the recently infected group and groups on days 5 and 8 p.i. are designed by *. Significant differences (p < 0.05) between the groups on days 5 and 8 p.i. are designed by #.
(DOCX)

S1 Video The subcapsular RP DC network in the spleen of a naïve mouse. Time-lapse images show a representative 3D region of the subcapsular RP of the spleen in a naïve B6.CD11c-YFP mouse. The video shows that most YFP^+^ cells (green) are sessile but are still actively making protrusions and extending dendrites. Images were acquired with Z-steps of 4 µm (total Z = 28 µm) for 30 min at 1 frame/19 s of live imaging. The video is shown at 10 frames/s with maximum color intensity.
(AVI)

S2 Video The uptake of iRBCs by subcapsular RP DCs soon after *Pc* infection. Time-lapse images show a representative 3D region of the subcapsular RP of the spleen in a B6.CD11c-YFP mouse, starting 15 min after i.v. infection with 1 × 10^8^ mature mCherry-*Pc* iRBCs. The video shows mCherry-*Pc* iRBCs (red) moving inside the RP and trapped by YFP^+^ cells (green). The white arrows indicate points of *Pc* trapping by YFP^+^ cells. Images were acquired with Z-steps of 4 µm (total Z = 28 µm) for 30 min at 1 frame/17 s of live imaging. The video is shown at 10 frames/s with maximum color intensity.
(AVI)

S3 Video Subcapsular RP DCs containing iRBC remnants soon after *Pc* infection. The animation shows a representative 3D region of the subcapsular RP of the spleen in a B6.CD11c-YFP mouse, starting 15 min after i.v. infection with 1 × 10^8^ mature mCherry-*Pc* iRBCs. The images show mCherry-*Pc* iRBC remnants inside YFP^+^ cells that are visualized as yellow spots of merged mCherry/YFP-3D signal. The video is shown at 10 frames/s with maximum color intensity.
(AVI)

S4 Video Trapping of iRBCs by subcapsular RP DCs soon after *Pc* infection. Time-lapse images show details from S2 Video in which a YFP^+^ cell (green) is in close contact with an mCherry-*Pc* iRBC (red). The parasite remained trapped in the YFP^+^ cell membrane throughout the video and moved from one part of the membrane to the other. The white arrows highlight this interaction between YFP^+^ cell and *Pc* parasite. The video is shown at 10 frames/s of with maximum color intensity.
(AVI)

S5 Video Trapping of iRBCs by F4/80^+^YFP^+^ and F4/80^-^YFP^+^ cells soon after *Pc* infection. The animation shows a representative 3D region of the subcapsular RP of the spleen in a B6.CD11c-YFP mouse that was injected i.v. with fluorescent anti-F4/80 mAbs, starting 15 min after i.v. infection with 1 × 10^8^ mature CMTPX-*Pc* iRBCs. The video shows YFP^+^ cells (green), iRBCs (red), F4/80^+^ cells (blue) and merged F4/80^+^YFP^+^ cells (white). The video is shown at 10 frames/s with maximum color intensity.
(AVI)

S6 Video CD4^+^ T cell / DC dynamics in the spleen of a naïve mouse. Time-lapse images show a representative 3D region of the subcapsular spleen in a B6.CD11c-YFP mouse adoptively transferred with polyclonal CD4^+^ T cells from B6.CFP mice. The video shows actively moving CD4^+^ T cells (blue) making non-stable and sporadic contacts with YFP^+^ cells (green). Images were acquired with Z-steps of 4 µm (total Z = 28 µm) for 60 min and are shown at 1 frame/15 s of live imaging. The video is shown at 10 frames/s with maximum color intensity.
(AVI)

S7 Video CD4^+^ T cell / DC dynamics in the spleen of a recently infected mouse. Time-lapse images show a representative 3D region of the subcapsular spleen in a B6.CD11c-YFP mouse adoptively transferred with polyclonal CD4^+^ T cells from B6.CFP mice, starting 24 h after i.v. infection with 1 × 10^8^ mature mCherry-*Pc* iRBCs. The video shows YFP^+^ cells (green) that are slightly more motile, still making protrusions and actively internalizing parasites (red). CD4^+^ T cells (blue) are less motile and are making more stable contacts with YFP^+^ cells. Images were acquired with Z-steps of 4 µm (total Z = 28 µm) for 60 min at 1 frame/15 s of live imaging. The video is shown at 15 frames/s with maximum color intensity.
(AVI)

S8 Video The uptake of iRBCs by subcapsular RP DCs during pre-crisis. Time-lapse images show a representative 3D region of the subcapsular RP of the spleen in a B6.CD11c-YFP mouse, starting 5 days after i.p. infection with 1 × 10^6^ mCherry-*Pc* iRBCs. The video was made at a time of day when mature parasite stages predominated and shows many mCherry-*Pc* iRBCs (red) inside the RP, as well as the intense phagocytic activity of YFP^+^ cells (green). The white arrows indicate points of *Pc* trapping by YFP^+^ cells. Images were acquired with Z-steps of 4 µm (total Z = 28 µm) for 30 min at 1 frame/19 s of live imaging. The video is shown at 15 frames/s with maximum color intensity.
(AVI)

S9 Video Phagocytosis of an iRBC by a subcapsular RP DC during pre-crisis. Time-lapse images show a detail from S8 Video in which a YFP^+^ cell (green) containing remnants of previous *Pc* internalizations (yellow spots of merged mCherry/YFP-3D signal) phagocytizes an mCherry-*Pc* iRBC (red). The video is shown at 15 frames/s with maximum color intensity.
(AVI)

S10 Video Subcapsular RP DCs containing iRBC remnants and in the process of phagocytizing an iRBC during pre-crisis. The animation shows a representative 3D region of the subcapsular RP of the spleen in a B6.CD11c-YFP mouse, starting five days after i.p. infection with 1 × 10^6^ mature mCherry-*Pc* iRBCs. The video was made at a time of day when mature parasite stages predominated, mCherry-*Pc* iRBC remnants inside YFP^+^ cells can be observed as yellow spots of merged mCherry/YFP-3D signal. It was possible to observe in 3D ongoing phagocytosis of an mCherry-Pc iRBC by a YFP^+^ cell. The white arrows indicate the ongoing phagocytosis described above. The video is shown at 10 frames/s with maximum color intensity.
(AVI)

S11 Video Trapping of iRBCs by F4/80^+^YFP^+^ and F4/80^-^YFP^+^ cells during pre-crisis. The animation shows a representative 3D region of the subcapsular RP of the spleen in a B6.CD11c-YFP mouse on day 5 p.i. that was injected i.v. with fluorescent anti-F4/80 mAbs, starting 15 min after i.v. injection with 1 × 10^8^ mature CMTPX-*Pc* iRBCs. The video shows YFP^+^ cells (green), iRBCs (red), few F4/80^+^ cells (blue) and merged F4/80^+^YFP^+^ cells (white). The video is shown at 15 frames/s with maximum color intensity.
(AVI)

S12 Video Subcapsular RP DCs containing iRBC remnants during crisis. The animation shows a representative 3D region of the subcapsular RP of the spleen in a B6.CD11c-YFP mouse, starting eight days after i.p. infection with 1 × 10^6^ mature mCherry-*Pc* iRBCs. The video was made at a time of day when mature parasite stages predominated. Images show mCherry-*Pc* iRBC remnants inside YFP^+^ cells as yellow spots of merged mCherry/YFP-3D signal. The white arrows indicate the few *Pc* parasites trapped by YFP^+^ cells. The video is shown at 10 frames/s with maximum color intensity.
(AVI)
