# Supplementary figures and images for: In Vivo Approaches Reveal a Key Role for DCs in CD4+ T Cell Activation and Parasite Clearance during the Acute Phase of Experimental Blood-Stage Malaria
Source: PLoS Pathog. 2015 Feb 6;11(2):e1004598. doi: 10.1371/journal.ppat.1004598 (PMC4450059; doi:10.1371/journal.ppat.1004598)

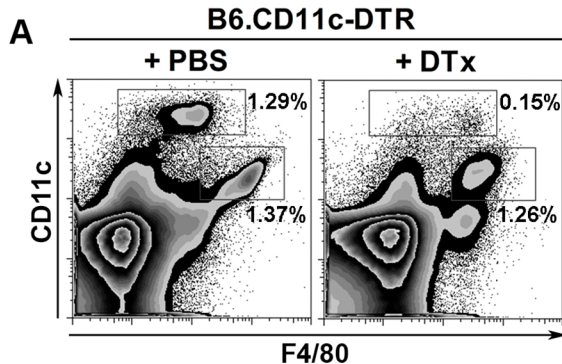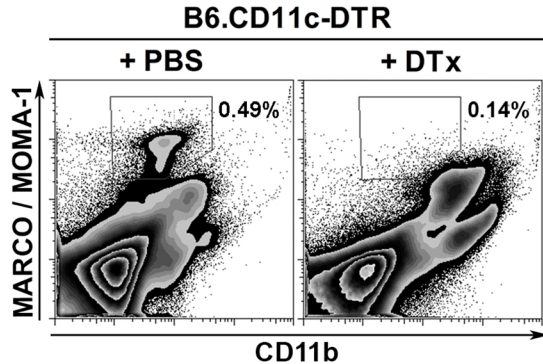

**B**

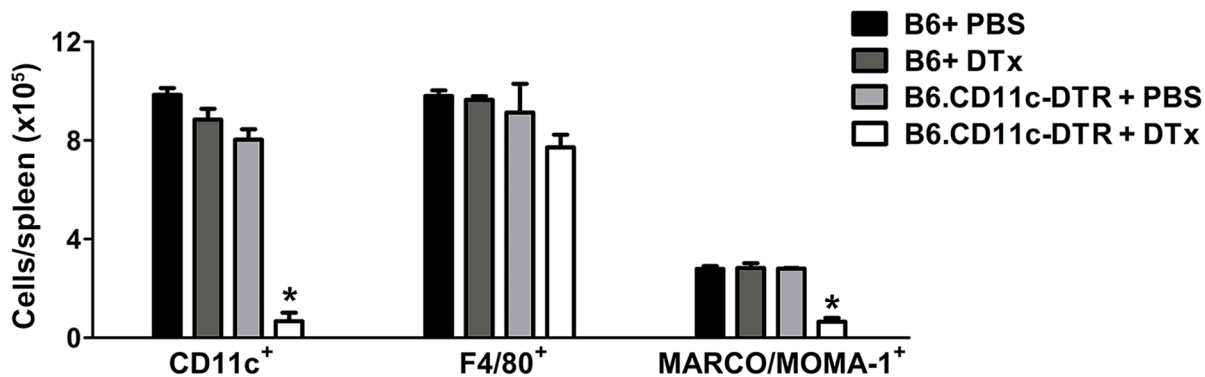

Supplement: S1 Fig — (A and B) B6 and B6.CD11c-DTR mice were treated with DTx or PBS, and their spleens were analyzed by flow cytometry after 24 h. (A) Representative contour plots show the depletion of CD11c+ and MARCO/MOMA-1+(CD11b+) cells, but not of F4/80+ cells, in DTx-treated B6.CD11c-DTR mice. Data show the percentages of CD11c+, F4/80+ and MARCO/MOMA-1+ cells in the splenocyte population. (B) The numbers of CD11c+, F4/80+ and MARCO/MOMA-1+ cells per spleen are shown. In B, significant differences (p < 0.05) between all other groups are designated by *. In A and B, one representative experiment out of three (n = 3) is shown. (PDF) [file ppat.1004598.s001.pdf]

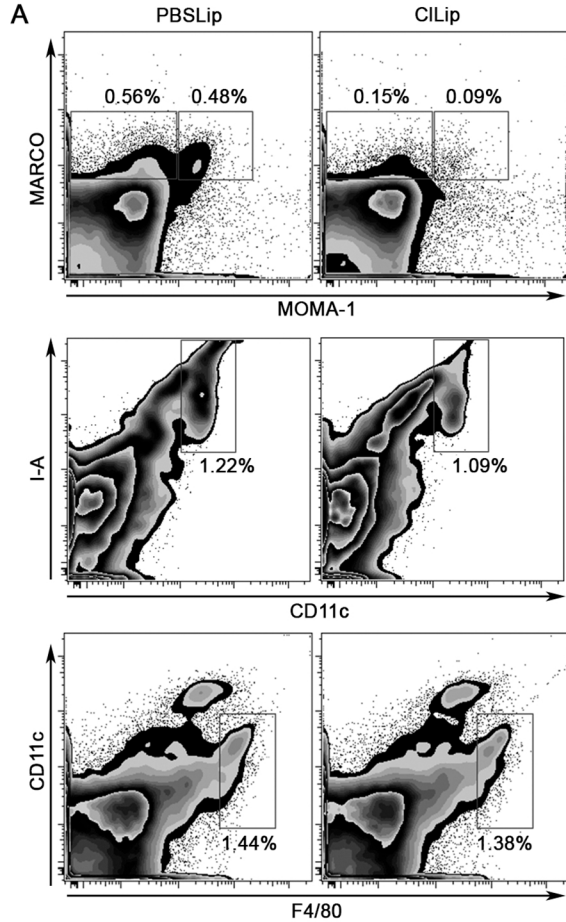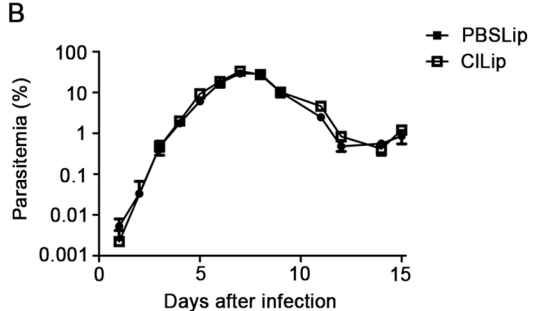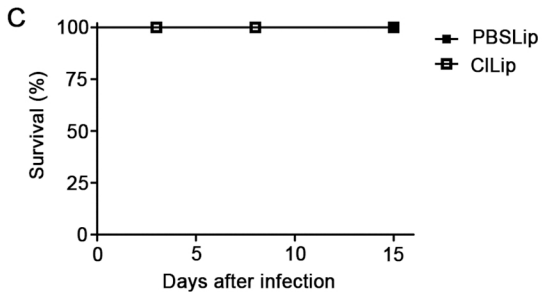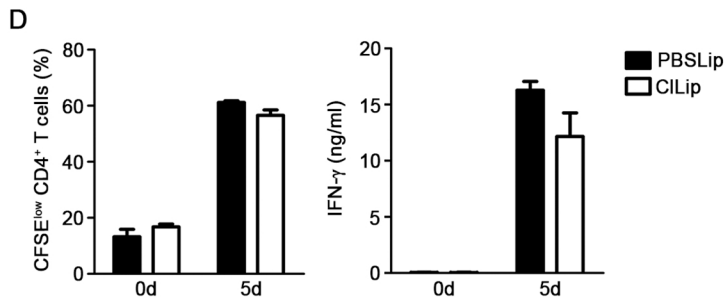

Supplement: S2 Fig — (A-D) B6 mice were treated with either a low dose of ClLip to deplete MARCO+ and MOMA-1+ macrophages or with PBSLip as controls. The mice were i.p. infected with 1 × 10^6^ Pc iRBCs 24 h later. (A) Representative contour plots obtained 24 h after treatment by flow cytometry confirm the efficiency of ClLip-induced depletion of MARCO+ and MOMA-1+ cells without affecting CD11c+I-A+ and F4/80+ cells. Data show the percentages of MARCO+, MOMA-1+, CD11c+I-A+ and F4/80+ cells in the splenocyte population. (B) Parasitemia curves are shown (means ± SD). (C) Survival curves are shown. (D) Data show the percentages of proliferating CFSElowCD4+ T cells and IFN-γ concentrations in the supernatants of spleen cell cultures stimulated for 72 h with iRBCs (means ± SD). In A-D, one representative experiment out of three (n = 5) is shown. (PDF) [file ppat.1004598.s002.pdf]

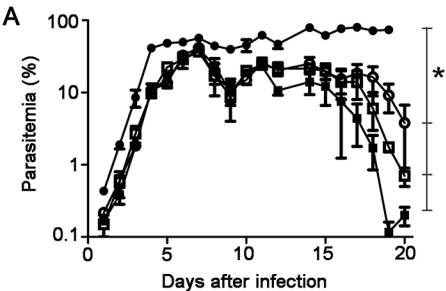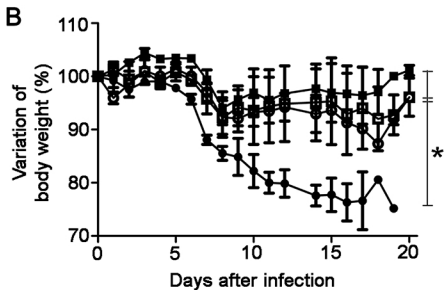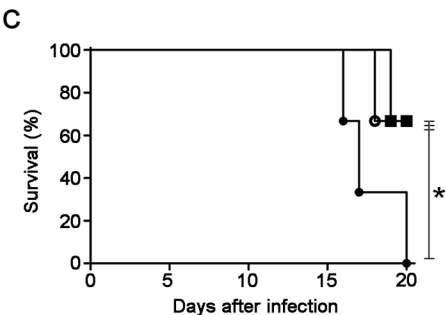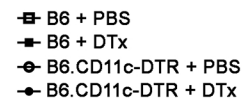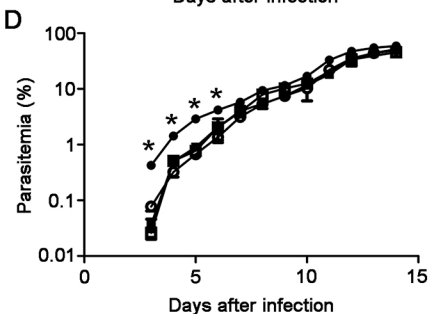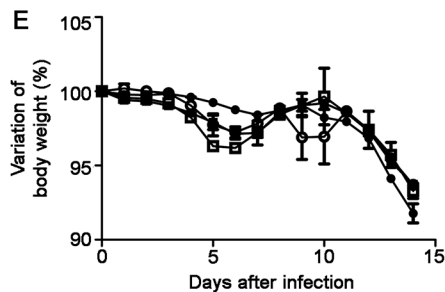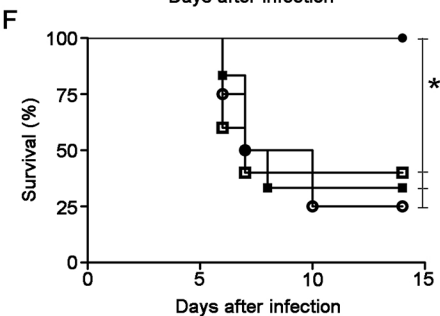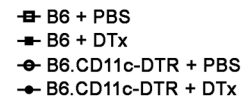

Supplement: S3 Fig — (A-C) B6 and B6.CD11c-DTR mice were treated with either DTx to deplete CD11c+ cells or PBS as a control. The mice were i.p. infected with 1 × 10^6^ Py iRBCs 24 h later. (A) Parasitemia curves are shown (means ± SD). (B) Variations in body weight relative to day 0 are shown (means ± SD). (C) Survival curves are shown. (D-F) B6 and B6.CD11c-DTR mice were i.v. infected with 1 × 10^3^ Pb sporozoites. After 48 h, the mice were treated with DTx to deplete CD11c+ cells at the beginning of blood stage. (D) Parasitemia curves are shown (means ± SEM). (E) Variations in body weight relative to day 0 are shown (means ± SEM). (F) Survival curves are shown. In A-F, significant differences (p < 0.05) between the indicated groups are designated by *. In A-C, one representative experiment out of three (n = 3-4) is shown. In D-F, data from three experiments (n = 2-3) are shown. (PDF) [file ppat.1004598.s003.pdf]

**A**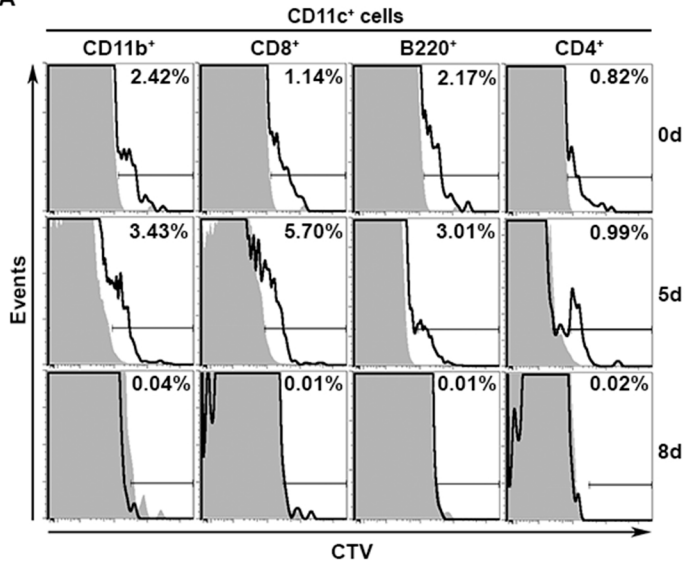**B**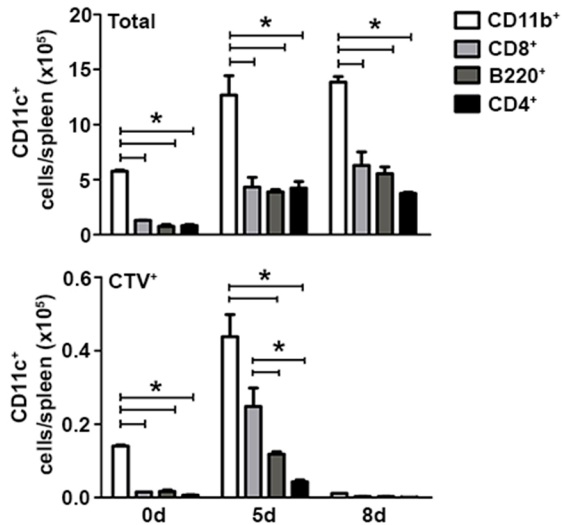

Supplement: S4 Fig — Spleens were analyzed 15 min after i.v. injection of 1 × 10^8^ mature CTV-Pc iRBCs (dark line histograms) or PBS (filled histograms) in B6 mice at zero, five or eight days p.i. with 1 × 10^6^ Pc iRBCs. (A) Representative histograms obtained by flow cytometry show CTV staining in the splenic DC subsets (CD11b+, CD8+, B220+ or CD4+). Data show the percentages of CTV+ cells in each subset. (B) Numbers of total and CTV+CD11c+ cells per spleen were calculated from the data obtained in A. In B, significant differences (p < 0.05) between the DC subsets at different days p.i. are designated by *. In A and B, one representative experiment out of three (n = 5) is shown. (PDF) [file ppat.1004598.s004.pdf]

**A****B6.CD11c-YFP**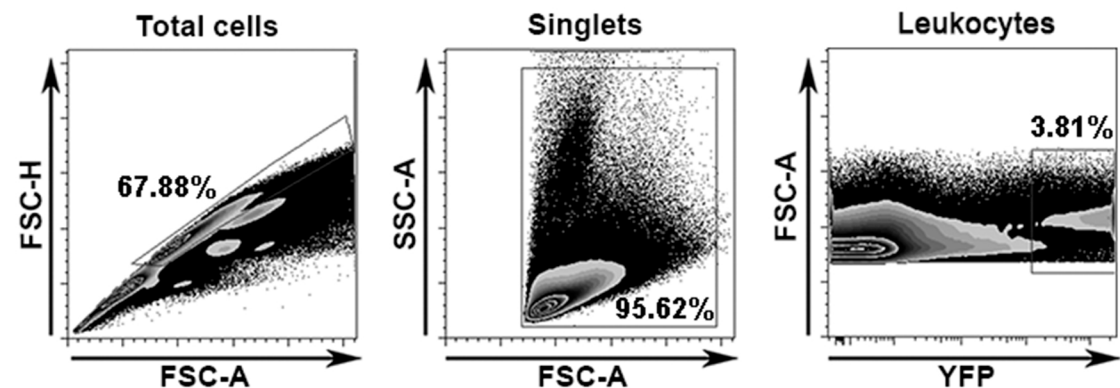**B****B6.CD11c-YFP, YFP<sup>+</sup> cells**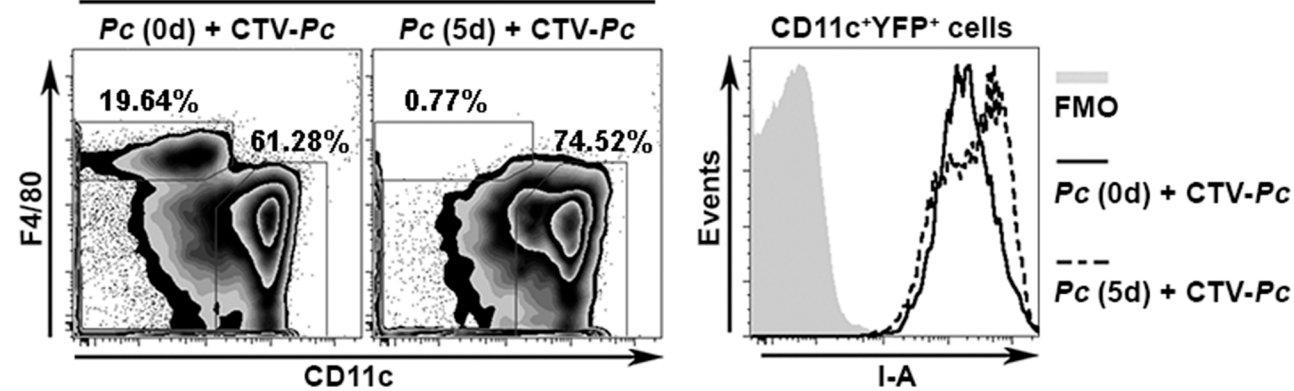

Supplement: S5 Fig — (A and B) Spleens were analyzed 15 min after i.v. injection of 1 × 10^8^ mature CTV-Pc iRBCs in B6.CD11c-YFP mice at zero or five days p.i. with 1 × 10^6^ Pc iRBCs. (A) Representative contour plots show the gate strategy for analysis of YFP+ cells in naïve mice. Data show the percentages of singlets, leukocytes and YFP+ cells in each contour plot. (B) Representative contour plots show CD11c and F4/80 staining in the YFP+ cells. Data show the percentages of these cells in the YFP+ cell population. Histograms show MHC class II (I-A) staining in CD11c+YFP+ cells. The fluorescence minus one (FMO) control was obtained in CD11c+YFP+ cells from a [Pc (0) + CTV-Pc] mouse (filled histogram). In A and B, one representative experiment out of three (n = 3) is shown. (PDF) [file ppat.1004598.s005.pdf]

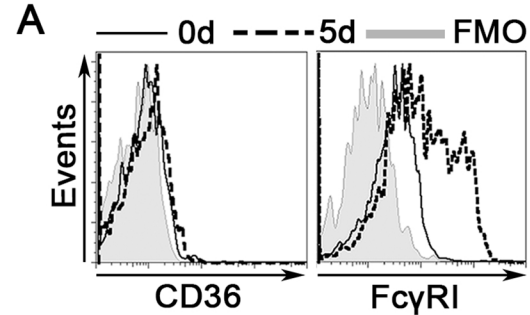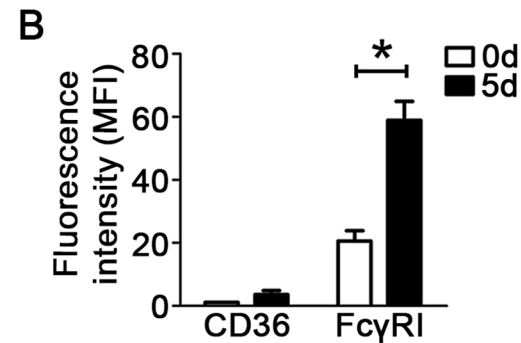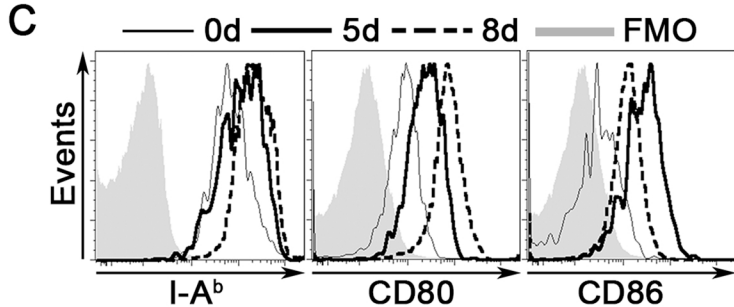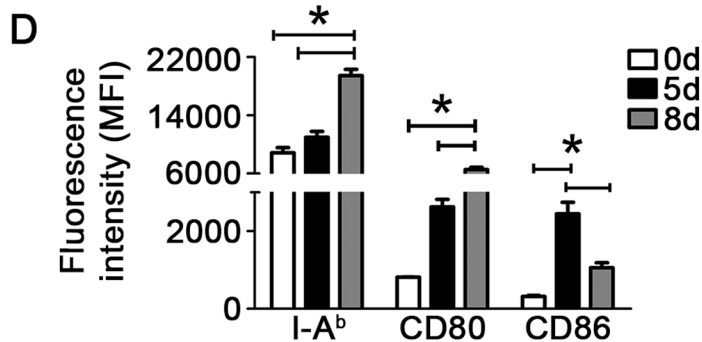

Supplement: S6 Fig — B6 mice were i.p. infected with 1 × 10^6^ Pc iRBCs. At zero, five or eight days p.i., spleens were analyzed by flow cytometry. (A) Representative histograms show the expression of CD36 and FcγRI in CD11c+ cells. The corresponding FMO control for each marker is represented by the filled histograms. (B) Median fluorescence intensity (MFI) was calculated from the data obtained in A (means ± SD). (C) Representative histograms show the expression of MHC class II (I-A), CD80 and CD86 molecules in CD11c+ cells. The corresponding FMO control for each marker is represented by the filled histograms. (D) MFI was calculated from the data obtained in C (means ± SD). In B and D, significant differences (p < 0.05) between the indicated groups are designated by *. In A-D, one representative experiment out of three (n = 5) is shown. (PDF) [file ppat.1004598.s006.pdf]
