# Supplementary material for: In Vivo Approaches Reveal a Key Role for DCs in CD4+ T Cell Activation and Parasite Clearance during the Acute Phase of Experimental Blood-Stage Malaria
Source: PLoS Pathog. 2015 Feb 6;11(2):e1004598. doi: 10.1371/journal.ppat.1004598 (PMC4450059; doi:10.1371/journal.ppat.1004598)
Supplement: S1 Table — The data compare several parameters in recently infected mice and mice on days 5 p.i. (pre-crisis) and 8 p.i. (crisis). For in vivo analyses, the following two experiments were performed: 1) B6.CD11c-YFP mice were i.v. infected with 1 × 10^8^ mature mCherry-Pc iRBCs, and spleens were evaluated 15 min later. 2) B6.CD11c-YFP mice were i.p. infected with 1 × 10^6^ mCherry-Pc iRBCs, and spleens were analyzed at five or eight days p.i., at a time of day when mature parasite stages predominated. For ex vivo analyses, the following two experiments were performed: 1) B6 mice were i.v. infected with 1 × 10^8^ purified mature CTV-Pc iRBCs (flow cytometry) or GFP-Pc iRBCs (flow cytometry and immunofluorescence), and spleens were evaluated 15 min later. 2) B6 mice were i.p. infected with 1 × 10^6^ GFP-Pc iRBCs (flow cytometry and immunofluorescence), and spleens were evaluated after five or eight days p.i., at a time of day when mature parasite stages predominated. Data were compiled from figs. 2, 3, 5 and 6. Significant differences (p < 0.05) between the recently infected group and groups on days 5 and 8 p.i. are designed by *. Significant differences (p < 0.05) between the groups on days 5 and 8 p.i. are designed by #. (DOCX) [file ppat.1004598.s007.docx]

**TABLE S1**

| PARAMETERS | TECHNIQUE | 15 min p.i. | 5 days p.i. | 8 days p.i. |
| --- | --- | --- | --- | --- |
| CD11c^+^ cell volume (µm^3^) (X 10^4^) | CIVM | 0.4±0.1 | 1.7±0,4*# | 0.3±0.1 |
| CD11c^+^ cell sphericity (a.u.) | CIVM | 0.7±0.1 | 0.6±0.1* | 0.6±0.1* |
| mCherry^+^CD11c^+^ cells (%) | CIVM | 15.9±2.3 | 44.6±6.2*# | 2.9±0.8* |
| CTV^+^CD11c^+^ cells (%) | Flow cytometry | 1.5±0.5 | 3.4±1.2*# | 0.1±0.0 |
| GFP^+^CD11c^+^ cells (%) | Flow cytometry | 2.4±0.8 | 11.4±3.8*# | 0.8±0.4 |
| CD11c-GFP pixel colocalization (%) | Immunofluorescence | 5.3±1.4 | 42.1±18.3* | _________ |
